# Betulin ameliorates neuronal apoptosis and oxidative injury via DJ‐1/Akt/Nrf2 signaling pathway after subarachnoid hemorrhage

**DOI:** 10.1111/cns.70019

**Published:** 2024-09-05

**Authors:** Xiaoyang Lu, Shu Yang, Qixiong Lu, Yuansheng Zhang, Zaihong Cha, Wei Huang, Tao Li

**Affiliations:** ^1^ Department of Neurosurgery, The First People's Hospital of Yunnan Province The Affiliated Hospital of Kunming University of Science and Technology Kunming Yunnan China; ^2^ Department of Neurology, The First People's Hospital of Yunnan Province The Affiliated Hospital of Kunming University of Science and Technology Kunming Yunnan China; ^3^ The Affiliated Hospital of Kunming University of Science and Technology. Department of Neurosurgery The First People's Hospital of Yunnan Province Kunming Yunnan China

**Keywords:** betulin, DJ‐1, early brain injury, neuronal apoptosis, oxidative injury, subarachnoid hemorrhage

## Abstract

**Aims:**

We aimed to resolve the uncertainty as to whether betulin exerted neuroprotection on early brain injury (EBI) caused by subarachnoid hemorrhage (SAH), and to investigate the related molecular mechanisms.

**Methods:**

Bioinformatic analysis was performed to pre‐study the differently expressed genes (DEGs) and the possible signaling pathways. Rat and cellular model of SAH were introduced in this study, and betulin, an activator of DJ‐1 protein, was administered to reveal the effect. Gross assessment regarding mortality, neurofunctions, SAH grade, brain water content (BWC) along with multiple cellular and molecular studies in vivo or/and in vitro such as immunofluorescence (IF) staining, western blot (WB), reactive oxygen species (ROS) assay, and flow cytometry (FCM) were all conducted after SAH induction to verify the protective effect and the relevant mechanisms of DJ‐1 in diverse levels. In addition, MK2206 (selective inhibitor of Akt) and iRNA*Dj‐1* (interfering RNA to *Dj‐1*) were utilized to confirm the mechanisms of the effect.

**Results:**

The data from our study showed that DJ‐1 protein was moderately expressed in neurons, microglia, and astrocytes; its level in brain tissue elevated and peaked at 24–72 h after SAH induction. Betulin could efficaciously induce the expression of DJ‐1 which in turn activated Akt and Bcl‐2, and anti‐oxidative enzymes SOD2 and HO‐1, functioning to reduce the activation of cleaved caspase‐3 (c‐Casp‐3) and reactive oxygen species (ROS). The induced DJ‐1 could upregulate the expression of Nrf2. However, Akt seemed no direct effect on elevating the expression of Nrf2. DJ‐1 alone could as well activate Akt‐independent antiapoptotic pathway via suppressing the activation of caspase‐8 (Casp‐8).

**Conclusions:**

Betulin which was a potent agonist of DJ‐1 had the ability to induce its expression in brain tissue. DJ‐1 had neuroprotective effect on EBI through comprehensive mechanisms, including facilitating intrinsic and extrinsic antiapoptotic pathway, and reducing oxidative injury by upregulating the expression of redox proteins. Betulin as an inexpensive drug showed the potential for SAH treatment.

## Graphical abstract

Schematic of betulin/DJ‐1/Akt/Nrf2 signaling pathway.

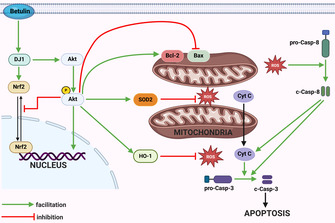

## 1 INTRODUCTION

Despite a profusion of scientific breakthroughs, subarachnoid hemorrhage (SAH) which is most frequently caused by the rupture of intracranial aneurysm remains a perilous issue clinically.^1^ It is reported that the mortality of SAH caused by single rupture can be up to 30%–50% and above, the patients who survive otherwise are mostly to suffer from neurological deficits lifelong.^2^ Numerous research programs therefore have been established to reveal the mechanisms of brain injury caused by SAH,^3^ expecting to dig out potential therapeutic targets for developing viable alternatives for clinicians to ameliorating the grievous prognosis of SAH. Although complete nature of SAH remains vague, shared pathological processes within its course have been cited by plenty of studies. Among the processes, early brain injury (EBI) which is comprised of several pathologies such as intracranial hypertension, cerebral vasospasm, perturbation to brain blood flow, brain edema, immune injury, oxidative overstress, neuronal apoptosis, neuroinflammation, and autophagy, has attracted the attention of researchers.^4^, ^5^, ^6^ Thus, one of the focuses of the research is to discover new signaling or metabolic pathways and the proteins involved, with the aim of designing chemical compounds to activate the inner protective mechanisms to alleviate neuronal injury, attaining the neuroprotection which might be of clinical practicality.

A novel oncogene originally identified in Parkinson's disease named *Park7* or *Dj‐1* is reported to be relative to autosomal‐recessive hereditary Parkinson's disease.^7^ Its protein product, namely DJ‐1, has been heatedly reported to show neuroprotective effect in not only neurodegenerative diseases^8^ but also many other neurological pathologies including cerebral stroke^9^ and traumatic brain injury.^10^ DJ‐1 is ubiquitously expressed in mammalian cells and moderately expressed in cells of central nervous system (CNS). It is redox sensitive and can be remarkably induced under oxidative stress.^11^ DJ‐1 functions as a neuroprotector in diverse properties, among which reducing mitochondrial oxidative stress by relocating from cytoplasm and nucleus to mitochondria has been regarded as the most essential one and thus studied repeatedly.^12^ Zeng et al. have reported that DJ‐1 stimulates antioxidative and antiapoptotic gene expression, promoting the pro‐survival Akt pathway.^13^ It is noted that DJ‐1 could be increasingly secreted under pathological circumstances like melanoma^14^ or breast cancer.^15^ Studies by Pantcheva et al. have found that other than intracellular protection, DJ‐1 plays its effect through paracrine and/or autocrine cues.^16^

Betulin, one chemical compound refined from *Betula platyphylla Suk* (birch tree), together with its derivatives has been broadly reported to have antiapoptotic,^17^ anticancer^18^, ^19^ and anti‐inflammatory effect.^19^ Zhou et al. have found that betulin can modulate immune response to alleviate liver damage from hepatitis.^20^ It is well reported that betulin's derivatives, betulinic acid (BA) has protective effect on virus and bacterial infection.^18^ Ci et al. reported in their study that betulin has the effect on regulating AMPK/AKT/Nrf2 signaling to mitigate inflammation.^21^

Based on these accumulating evidence and results, we therefore presumed that DJ‐1 had same merits in EBI caused by SAH via regulating Akt/Nrf2 signaling pathway, for kindred mechanisms play similar roles in multiple neurological diseases, and subsequently initiated our study utilizing betulin to explore its impact on DJ‐1 and the possible effect on Akt/Nrf2 signaling pathway and the outcome of SAH.

## 2 MATERIALS AND METHODS

### 2.1 Bioinformatic analysis

The publicly available RNA‐Seq GSE79416 used in this study were obtained from GEO using the R package GEOquery.^22^ Matrix data were processed using RStudio (R version 4.3.2).^23^, ^24^ To detect subtle changes in pathway activity in samples of expression datasets, we used GSVA.^25^ We downloaded pathway datasets from the Molecular Signatures Database (MSigDB) (version 7.4)^26^ and AmiGO.^27^ ssGSEA was performed on matrices of RNA‐Seq samples with reference to hallmark gene sets. Statistical significance was determined using Student's unpaired two‐tailed *t*‐test. We considered a *p*‐value less than 0.05 statistically significant. Graphs and numbers were generated using the R packages ggplot2 (version 3.4.4), ggpubr (version 0.6.0), and ggsci (version 3.0.0).

### 2.2 Study in vivo

#### 2.2.1 Animals

Male Sprague–Dawley rats (290‐310 g, 300 ± 10 g) were purchased from SLAC Laboratory Animal Co., Ltd. (Shanghai, China) and housed in a strictly controlled environment with a 12 h dark cycle and of foraging for food and water ad libitum. All procedures regarding experimental animals were referred to the National Institutes of Health (NIH) Guide for the Care and Use of Laboratory Animals, and approved and supervised by the Committee of Experimental Animal Ethics, Kunming University of Science and Technology (Permission no. 2020YJC137).

#### 2.2.2 SAH model in vivo

The endovascular perforation model was adopted for the study, as it is closer to the natural course of SAH caused by intracranial aneurysmal rupture. The model establishment began with anesthesia. Precisely, rat was anesthetized with pentobarbital (40 mg/kg) injection peritoneally (*ip*.). Next, the rat was immobilized in a supine position, the anatomy of the neck was performed with necessary exposure of left carotid artery (CA) and its branches, then a 4‐0 sharpened nylon suture was inserted in external carotid artery (ECA) which was dissociated beforehand, and propelled along internal carotid artery (ICA) toward the intracranial bifurcation of middle cerebral artery (MCA) and anterior cerebral artery (ACA). Not until the greatest resistance was met did the suture come to a halt at the bifurcation which was commonly the perforating spot to initiate SAH. A grading system was employed to quantitatively evaluate and rate the severity of SAH. That was, by dividing the basal cistern into six segments, and each segment was scored from 0 to 3, where 0 meant no subarachnoid blood and 3 represented all arteries within the segment being covered by blood clot. Additionally, Score 1 represented minimal subarachnoid blood and 2 stood for moderate blood with visible arteries. Having scores added from six segments to make a total score which was ranging from 0 to 18, the severity of SAH could be eventually rated: mild SAH (0–7), moderate SAH (8–12), and severe SAH (13–18). The brain tissue collected and used for experiments in this study was from the cerebral cortex ipsilaterally to the puncture side.

#### 2.2.3 Drug administration

Betulin (Sigma‐Aldrich, B9757) was dissolved in dimethyl sulfoxide (DMSO, 5%) to create a solution with the concentration of 20, 40, and 80 mg/kg, respectively, based on the recommendation of manufacturer's instruction and the literature.^18^, ^28^ The groups receiving betulin treatment were given the solution peritoneally (*ip*.) at 1 h after SAH induction, while the vehicle group was given the same volume of DMSO (*ip*.). The minimal‐effective dosage was adopted for the following experiments.

#### 2.2.4 Mortality, SAH score, and Neurological score

The research cross‐section was designed at 24 and 72 h after the induction of SAH. Mortality was calculated, SAH grade was determined using Sugawara method,^29^ and neurological assessment was conducted on the basis of Garcia scoring system^30^ then. It was a modified scoring system for neurofunctions of the rodent which took account of six aspects together: spontaneous activity, symmetry of four‐limb movement, forelimb outstretching, climbing, body proprioception, and the response to vibrissae touch. By adding up the scores of each single test varying from 0 to 3, a total score in the range of 0–18 could be gained for the assessment of systemic neurofunctions, where 0 was the worst and 18 represented an intact neurofunction. All tests, calculations, and assessment were conducted by a separate staff member.

#### 2.2.5 Brain water content

Brain water content (BWC) was quantified as previously described.^2^ Briefly, the euthanized rat had the brain dissected without perfusion. The brain was weighed to get the wet weight (WW) and then dried in constant temperature (105°C) for 24 h to get the dry weight (DW). BWC = [(WW‐DW)/WW × 100%]. BWC is a direct way to assess the severity of brain edema.

#### 2.2.6 Measurement of reactive oxygen species

The level of reactive oxygen species (ROS) in samples from rat brain was determined using a ROS assay kit (E004, Nanjing Jiancheng Bioengineering Institute, Nanjing, China) based on the manufacturer's protocol. To be brief, a suspension containing separated cells was mixed with DCFH‐DA (2,7‐dichlorofluorescin diacetate) solution which was 10 μM in concentration to yield a mixture (1: 1000). The mixture was then incubated at 37°C for 30 min before the centrifugation at 2000 revolution per minute (RPM) for 5 min. After having the supernatant discarded and adding in PBS (phosphate buffered saline, 0.01 M, 4°C, pH 7.40), the suspension was then centrifuged under the same condition. The final suspension was examined under light beams having excitation wavelength of 480 nm and emission wavelength of 520 nm with a spectrofluorophotometer. The level of ROS was interpreted as fluorescence intensity per gram of proteins.

#### 2.2.7 Mid‐ and long‐term neurofunction

Mid‐term (1–3 weeks) neurofunction was assessed using rotarod test.^31^ The rotating speed was fixed at 5 RPM and 10 RPM, and the stable time duration was recorded. In terms of long‐term (3–4 weeks) neurofunction assessment, Morris water maze was utilized.^31^ In brief, the device of Morris water maze was comprised with a circular pool, platform, and the tracking system. Before the test, nontoxic dark dye was poured into the water (25°C), and all rats should be tested at the baseline. After SAH induction, the platform was placed in the pool with a clockwise or counterclockwise order and the rat to be tested should be released at the farthest block (quadrant) to the target block. This test was designed for motor and learning function and began at 21 days after SAH and lasted for 6 days.

#### 2.2.8 Immunofluorescence staining

Immunofluorescence (IF) staining likewise as described,^31^ at 24 h after SAH induction, rats were anesthetized to undergo a transcardial perfusion with PBS (0.01 M, 4°C, pH 7.40), followed by another perfusion with a paraformaldehyde (PFA) solution (4%, 4°C, pH 7.40). The brain specimen was harvested and then preserved in same PFA solution at 4°C for fixation for 48 h. Brain specimens processed were available to be refrigerated in frozen medium for further histocytological study. Each frozen specimen was sectioned into 10 μm sections which were then mounted on a glass slide. The mounted sections to be performed with the immunofluorescence stain were initially rinsed using PBS (0.01 M, pH 7.40) for several times, then a blocking solution was added on the sections for the blockage (2 h, 25°C) of nonspecific bindings. The resultant slides were first incubated with primary antibodies: anti‐NeuN (1:500, Abcam ab177487), anti‐Iba‐1 (1:500, Wako 019–19,741), anti‐GFAP (1:500, Abcam ab7260), and anti‐DJ‐1 (1:1000, Abcam ab18257), for 12 h at 4°C. When the overnight incubation was finished, the slides were rinsed to remove the excess antibodies for the second incubation (2 h, 25°C) with corresponding secondary antibodies in a dark environment. The DAPI staining was used to seal the slides and mark the cell nucleus. All stained slides were examined under a fluorescence microscope (Olympus, Tokyo, Japan) by independent researchers. For cell count, five slides from the same brain specimen shall each have four different fields (200×) of vision examined, focusing mainly on the nearby areas of the perforating spot on the left cortex.

### 2.3 Study in vitro

#### 2.3.1 Cell culture and SAH model in vitro

The human neuroblastoma SH‐SY5Y cells were cultured (37°C, 5% CO_2_) in a DMEM/F12 medium containing 10% fetal bovine serum (FBS) in microplates. To establish the SAH model in vitro, OxyHb (10 μM) was introduced into the medium to co‐incubate (37°C, 5% CO_2_) with the cells for 24 h. When the incubation finished, the medium was removed and the cells were washed with PBS (0.01 M, pH 7.40) twice before any further studies. The treatment group received 20 μM betulin^32^ whereas the vehicle group had equal volume of DMSO (0.1%).

#### 2.3.2 RNA interference in vitro

The SY5Y cells were seeded onto 24‐well plates to be of 60%–80% consistency. According to the manufacturer's instructions, the transfection was performed by introducing iRNA*Dj‐1* assay (ThermoFisher, Assay ID 193132, 193133, and 193134) into the cell culture to co‐incubate at 37°C for 72 h. Detailed steps of the transfection and the sequences of iRNA*Dj‐1* involved were documented in supplemental files—Data S1.

#### 2.3.3 Measurement of cell viability: MTT assay

The 3‐(4,5‐dimethylthiazol‐2‐yl)‐2,5‐diphenyltetrazolium bromide (MTT) assay kit was utilized to measure the cell viability according to the instructions from the manufacturer. Briefly, the SH‐SY5Y cells should be cultured in 96‐well plates at a cell density ranging from 1 × 10^4^/well to 5 × 10^4^/well with culture medium measuring 100 μL per well. The cells in each well were treated with 10 μL of MTT solution (5 mg MTT in 1 mL 0.01 M PBS, pH = 7.40) before being incubated at 37°C for 2 h. After incubation, having the medium discarded and then added 100 μL of DMSO in each well to dissolve insoluble formazan. The cell viability was thereafter determined by measuring the absorbance at a wavelength of 570 nm using a microplate reader.

#### 2.3.4 Flow cytometry

The 12‐well plates holding SH‐SY5Y cells should first have the culture medium removed, then added with EDTA‐free trypsin (0.25%) to detach the cells. Second, a dilute solution of FBS (0.01 M PBS containing 10% FBS) was applied to the turbid liquid to terminate the reaction. The resultant mixture was centrifuged at 2000 RPM for 5 min, followed by a removal of the supernatant and the wash with PBS (0.01 M, pH = 7.40, 4°C). After repeating the process once, a buffer solution (200 μL) was introduced into it to resuspend the cells. The annexin A5 labeled with fluorescein isothiocyanate (Annexin‐V‐FITC, 5 μL) was added to the cell suspension for the first incubation (4°C, 15 min) in a dark environment, followed by the addition of propidium iodide (PI, 1 μL) for the re‐incubation in identical conditions. At the conclusion of incubation, an additional buffer solution (300 μL) was added, and the mixture should be analyzed shortly using a flow cytometer.

### 2.4 Study both in vivo and in vitro

#### 2.4.1 Western blot

Cortical tissue or SY5Y cells were fractionated thoroughly using a homogenizer and cell lysate to yield a mixture, which was then centrifuged at 12,000 RPM for 10 min at 4°C. The resultant supernatant was drawn out for further centrifugation, and the final supernatant containing whole proteins was extracted for the accurate determination of protein concentration using an assay kit (Bio‐Rad, Hercules, CA, USA). The calibrated protein solution was eventually resuspended in loading buffer, denatured at 95°C for 5 min, to yield standardized protein samples (40 μg/10 μL). Equal amount of the protein samples (10 μL) was loaded in the wells of polyacrylamide gel buffered with sodium dodecyl sulfate (SDS) for the following SDS polyacrylamide gel electrophoresis (SDS‐PAGE). When satisfactory separation reached, proteins within the gel were then transferred to a polyvinylidene difluoride (PVDF) membrane. Not until the rigorous transference of target proteins was completed did it ceased running. The membrane must be placed in protein solution (10% nonfat milk) for not less than 1 h to block nonspecific bindings before any further processes. After blocking, the membrane was incubated with a dilute solution (5% nonfat milk) of primary antibodies: anti‐DJ‐1 (1:10000, Abcam ab18257), anti‐p‐Akt (1:2000, CST #4060), anti‐Akt (1:1000, CST #4685), anti‐Bcl‐2 (1:500, Abcam ab59348), anti‐Bax (1:1000, Abcam ab32503), anti‐cleaved Caspase‐3 (1:500, Abcam ab13847), anti‐cleaved Caspase‐8 (1:1000, Abcam ab25901), anti‐SOD2 (1:2000, Abcam ab68155), anti‐HO‐1(1:20000, Abcam ab68477), anti‐Nrf2 (1:1000, Abcam ab62352), anti‐GAPDH (1:20000, CST #2118), and anti‐β‐actin (1:5000, Abcam ab8226) at 4°C overnight. The probed membrane was rinsed to remove excess antibodies, and in the meantime the corresponding secondary antibodies were diluted with 5% nonfat milk for the second incubation (25°C, 1 h). Detection and quantification of proteins could be conducted by analyzing the greyscale of bands on the membrane with the aid of a gel documentation system (Gel Doc XR^+^, Bio‐Rad) and an image processing software (ImageJ, NIH). The blotting for rat brain tissue was performed as the previous description and the details of protein extraction from the cell culture.^2^

### 2.5 Experimental study design

#### 2.5.1 Experiment 1: Time course of the amount of DJ‐1 and the research cross‐section (*n* = 40)

The fluctuation in the amount of DJ‐1 in brain tissue over time after SAH induction was studied, the research cross‐section was to be decided at the point‐in‐time in the time course where a peak or a nadir was reflected. In total, 36 rats were randomly allotted to six groups: sham (*n* = 6), SAH 3 h (*n* = 6), SAH 6 h (*n* = 6), SAH 12 h (*n* = 6), SAH 24 h (*n* = 6), and SAH 72 h (*n* = 6). For each group, the rats had cortical tissue harvested for protein analysis using WB. Based on the result of the time course, brain slides from Sham group (*n* = 2) and SAH 24 h group (*n* = 2) were applied to the IF staining.

#### 2.5.2 Experiment 2: Effect of betulin and the oxidative stress (*n* = 144)

We explored the effect of betulin and the most appropriate dosage. A total of 120 rats were randomly selected and allocated to five groups: sham (*n* = 6), vehicle (DMSO, *n* = 6), betulin (20 mg/kg, *n* = 6), betulin (40 mg/kg, *n* = 6), and betulin (80 mg/kg, *n* = 6). Apart from perforation and sham injection, rats in other four groups received the entirely same procedures. The systemic evaluation including mortality rate, SAH grade, Garcia test, brain edema, and oxidative stress at 24 and 72 h after SAH induction was implemented. The rotarod and Morris water maze tests was performed to evaluate the mid‐ and long‐term neurofunctions by establishing sham (*n* = 8), vehicle (*n* = 8), and betulin (*n* = 8) groups.

#### 2.5.3 Experiment 3: Mechanism of betulin's effect (*n* = 24)

We launched a thorough inquiry into the mechanisms of the effect of betulin, by virtue of MK2206 and iRNA*Dj‐1*.

1. In the first step of Experiment 3, we formulated four groups of rats: sham (*n* = 6), SAH (*n* = 6), SAH + vehicle (*n* = 6), and SAH + MK2206 (*n* = 6). To begin with, we compared these four groups in a study to detect the expression of DJ‐1, p‐Akt/Akt, and cleaved caspase‐3 (c‐Casp‐3). Next, we employed four groups (sham (n = 6), vehicle (*n* = 6), SAH + betulin (*n* = 6), and SAH + betulin+MK2206 (*n* = 6)) in another study to compare the quantity of DJ‐1, c‐Casp3, p‐Akt/Akt, Bcl‐2/Bax, cleaved caspase‐8 (c‐Casp8), SOD2, HO‐1, and Nrf2.
2. The second step of Experiment 3 was to verify the mechanisms and pathways in vitro with the addition of iRNA*Dj‐1*. Likewise, we organized a set of five groups: control (*n* = 6), vehicle (*n* = 6), betulin (*n* = 6), betulin+MK2206 (*n* = 6), and betulin+iRNA (*n* = 6), using WB to check the content of DJ‐1, p‐Akt/Akt, SOD2, HO‐1, and Nrf2.

Additionally, MTT assay and FCM were performed to calculate the cell viability and apoptotic rate to further confirm the effect of betulin and the mechanisms.

### 2.6 Statistical analysis

All the statistical data were pretested with distribution patterns using Shapiro–Wilk (SW) test and Kolmogorov–Smirnov (KS) test. Data which were normally distributed were calculated using one‐way analysis of variance (ANONA), and the results were presented as mean ± SD. Those were not in accord with normal distribution were processed with nonparametric test, and the results were presented as quartile. By comparing the difference between every two groups of data, when the *p*‐value was less than 0.05 (*p* < 0.05), it was recognized as statistically significant.

## 3 RESULTS

### 3.1 RNA‐seq data analysis and visualization

We have analyzed the publicly available RNA‐Seq dataset GSE79416, which comprised data on six brain cortex samples of normal mice and SAH mice. GSVA of hallmark gene sets from MSigDB revealed that the apoptosis and reactive oxygen species pathway were upregulated after SAH (Figure S1A). According to Gene Ontology analyses, the response to reactive oxygen species, apoptotic process, inflammatory cell apoptotic process, and positive regulation of apoptotic process were significantly increased following SAH (Figure S1B). We selected 10 genes related to DJ‐1 and its downstream signaling pathways, including Park7 (DJ‐1), Nfe2l2 (Nrf2), Akt1, Sod2, Hmox1 (HO‐1), Bcl2, Bax, Casp8, and Casp3. Most of these 10 genes are expressed highly in the SAH group samples (Figure S2).

#### 3.1.1 Mortality rate

No rat in sham group was deceased (0/46) while no differences were found between all the SAH groups. The general death rate in SAH groups was 6.4% (11/173). The detailed rat grouping and mortality rate can be referred to in Supplemental File 2—Data S1.

#### 3.1.2 Time course and distribution of DJ‐1 in brain tissue

**FIGURE 1:**
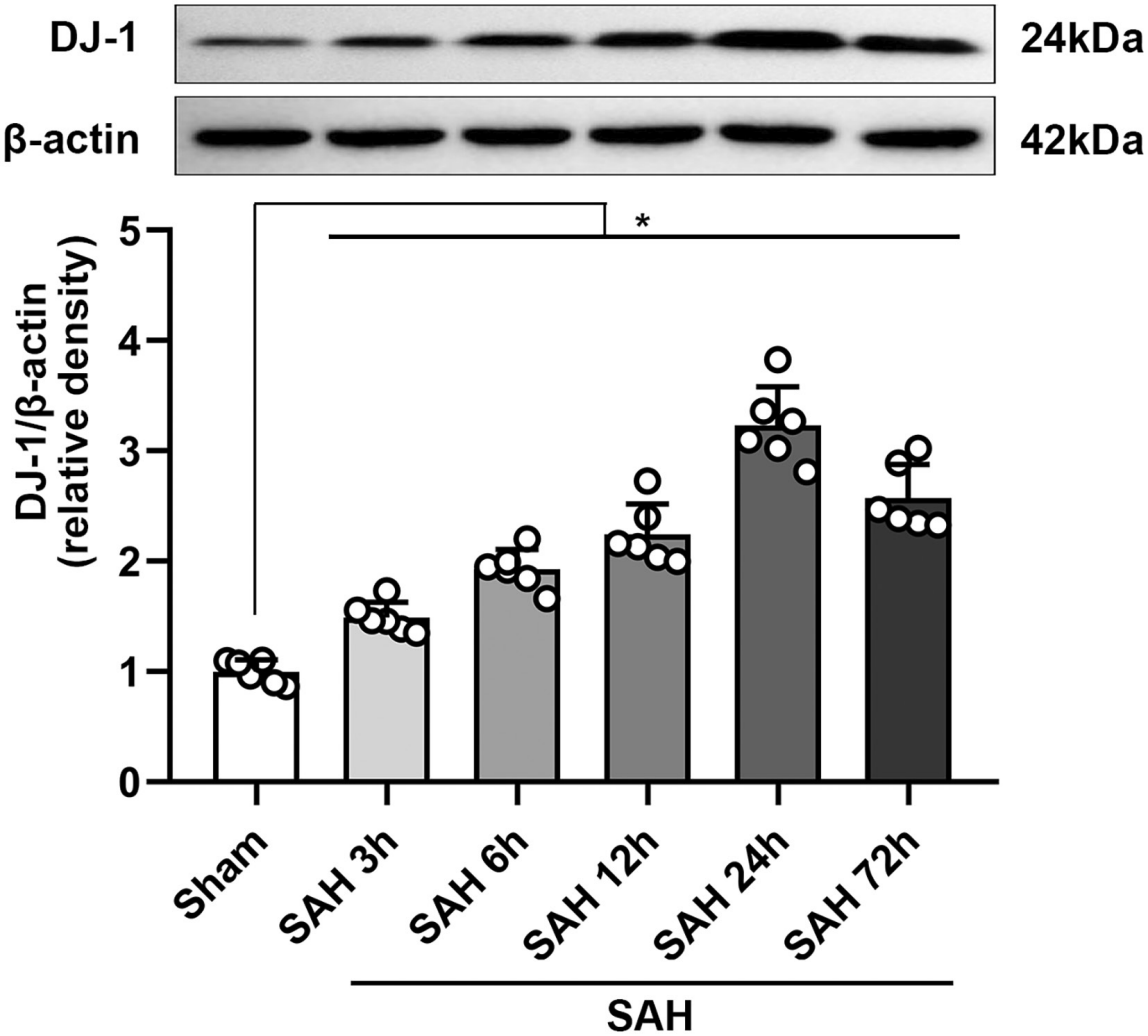
Expression of DJ‐1 before and after SAH detected using WB. Representative bands of WB and analysis showing that the expression level increased significantly 3 h after SAH and reached the apex at 24–72 h. One‐way ANOVA, *p<0.05 versus sham, (*n* = 6/group).

**FIGURE 2:**
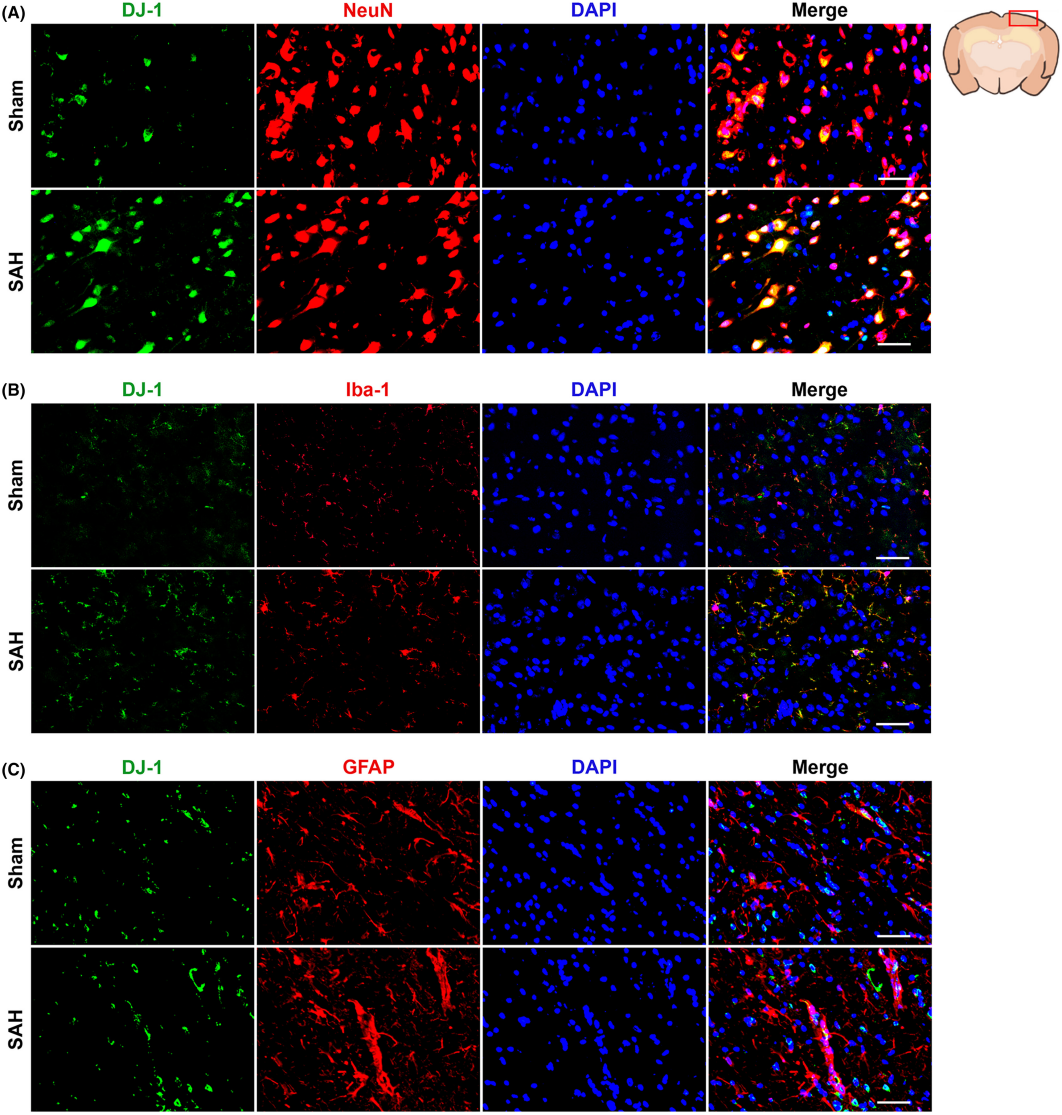
Expression and distribution of DJ‐1 in brain tissue. DJ‐1 (green) is expressed moderately in (A) neurons (red), (B) microglia (red), and (C) astrocytes (red) and increased after SAH (*n* = 2/group).

The expression of DJ‐1 could be noted to increase significantly 3 h after SAH and appeared to reach an apex at 24–72 h (Figure 1). Therefore, it is reasonable to choose the time point at 24 h to be the research cross section for the following studies. Through IF staining, we observed the increase in the expression of DJ‐1 after SAH and the moderate distribution of DJ‐1 in neurons, microglia, and astrocytes (Figure 2).

#### 3.1.3 Assessment at acute stage of SAH (24–72 h)

**FIGURE 3:**
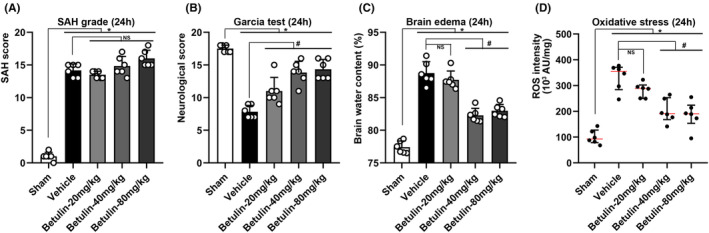
Gross assessment at 24 h after SAH. (A) SAH score surged after SAH induction; however, no significance was found between vehicle group and betulin treatment groups, one‐way ANOVA, *p < 0.05 versus sham, #*p* < 0.05 versus vehicle, (*n* = 6/group). (B) Neurological score dropped significantly after SAH, and the neurofunction was improved by using betulin, one‐way ANOVA, *p < 0.05 versus sham, #*p* < 0.05 versus vehicle, (*n* = 6/group). (C) BWC increased significantly after SAH while administration of betulin could alleviate the brain edema effectively, one‐way ANOVA, *p < 0.05 versus sham, #*p* < 0.05 versus vehicle, (*n* = 6/group). (D) ROS intensity grew significantly after SAH, indicating a remarkable oxidative injury; intervention with betulin significantly lessened the oxidative stress, nonparametric test, *p < 0.05 versus sham, #*p* < 0.05 versus vehicle, (*n* = 6/group). NS means statistical insignificance.

**FIGURE 4:**
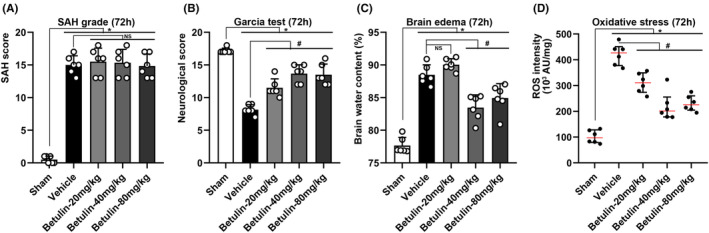
Gross assessment at 72 h after SAH. (A) SAH score surged after SAH induction; however, no significance was found between vehicle group and betulin treatment groups, one‐way ANOVA. (B) Neurological score dropped significantly after SAH, and the neurofunction was improved by using betulin, one‐way ANOVA. (C) BWC increased significantly after SAH while administration of betulin could alleviate the brain edema effectively, one‐way ANOVA. (D) ROS intensity grew significantly after SAH, indicating a remarkable oxidative injury; intervention with betulin significantly lessened the oxidative stress, nonparametric test. *p < 0.05 versus sham, #*p* < 0.05 versus vehicle, (*n* = 6/group). NS means statistical insignificance.

SAH score in sham group was near zero while surged significantly after SAH. However, there was no significance found among the vehicle and betulin groups, suggesting that betulin administration could not change the severity of SAH once it occurred (Figure 3A). The neuroscore dropped (*p* < 0.05, Figure 3B) whereas BWC (*p* < 0.05, Figure 3C) and ROS intensity (*p* < 0.05, Figure 3D) mounted up significantly after SAH; the rats treated with betulin showed an improvement in neurofunction and mitigated brain edema and oxidative stress at 24 h after SAH, especially on the medium‐ (40 mg/kg) and high‐dose (80 mg/kg) groups (*p* < 0.05, Figure 3B–D). Similar results were exhibited 72 h after SAH, indicating that though betulin did not reverse the course of SAH (Figure 4A) it could effectively refine the neurological outcome (*p* < 0.05, Figure 4B), brain edema (*p* < 0.05, Figure 4C), and oxidative injury (*p* < 0.05, Figure 4D).

#### 3.1.4 Mid‐ and long‐term neurofunction

**FIGURE 5:**
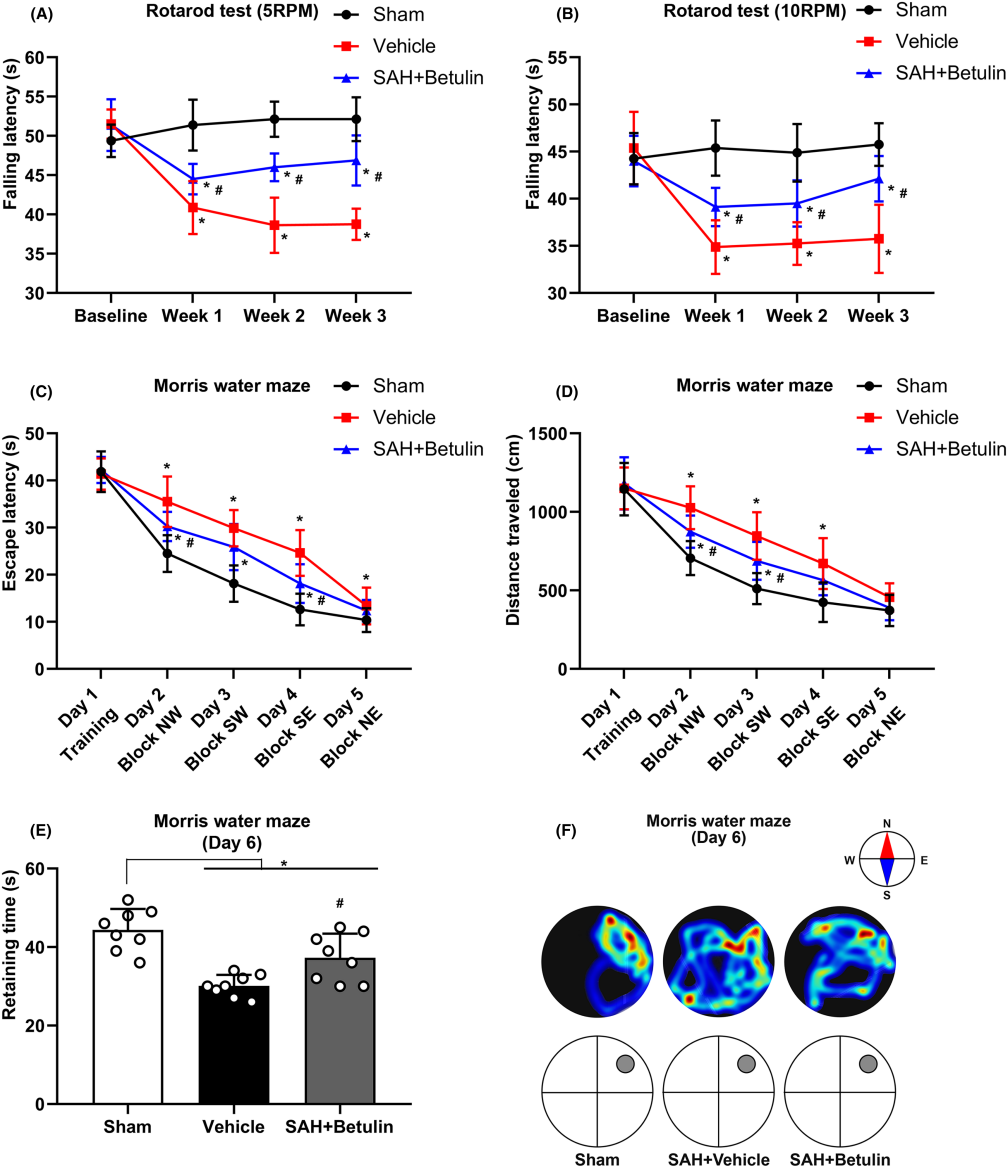
Mid‐ and long‐term neurofunction test. Fixed rotarod test at the rotating speed of (A) 5RPM and (B) 10RPM suggested that there was no difference on the baseline, but the falling latency decreased significantly after SAH; the use of betulin could reverse the neurofunctional deficits. Two‐way ANOVA, *p < 0.05 versus sham, #*p* < 0.05 versus vehicle, (*n* = 8/group). Morris water maze test showed that (C) escape latency and (D) distance traveled increased significantly after SAH and could be rescued by betulin, two‐way ANOVA, *p < 0.05 versus sham, #*p* < 0.05 versus vehicle, (*n* = 8/group). (E) Time in target quadrant fell significantly, and it was increased by using betulin, one‐way ANOVA, *p < 0.05 versus sham, #*p* < 0.05 versus vehicle, (*n* = 8/group). NS means statistical insignificance. (F) Heatmap displayed a straightforward picture, indicating that betulin could ameliorate the motor and learning function. Betulin dose of 40 mg/kg was used in this study.

Results of rotarod test showed no statistical difference at baseline for all rats; however, the falling latency at 5RPM (*p* < 0.05, Figure 5A) and 10RPM (*p* < 0.05, Figure 5B) decreased significantly after SAH induction. The group treated with betulin had a significant rise in falling latency, indicating betulin treatment could significantly improve the mid‐term neurological outcome of SAH in rats. Parameters including escape latency (*p* < 0.05, Figure 5C), distance traveled (*p* < 0.05, Figure 5D), and retaining time in target quadrant (*p* < 0.05, Figure 5E) of Morris water maze presented similar results, and the heatmap (Figure 5F) displayed a straightforward picture, which all suggest that betulin treatment could also ameliorate the long‐term outcome.

#### 3.1.5 Antiapoptotic effect of DJ‐1

**FIGURE 6:**
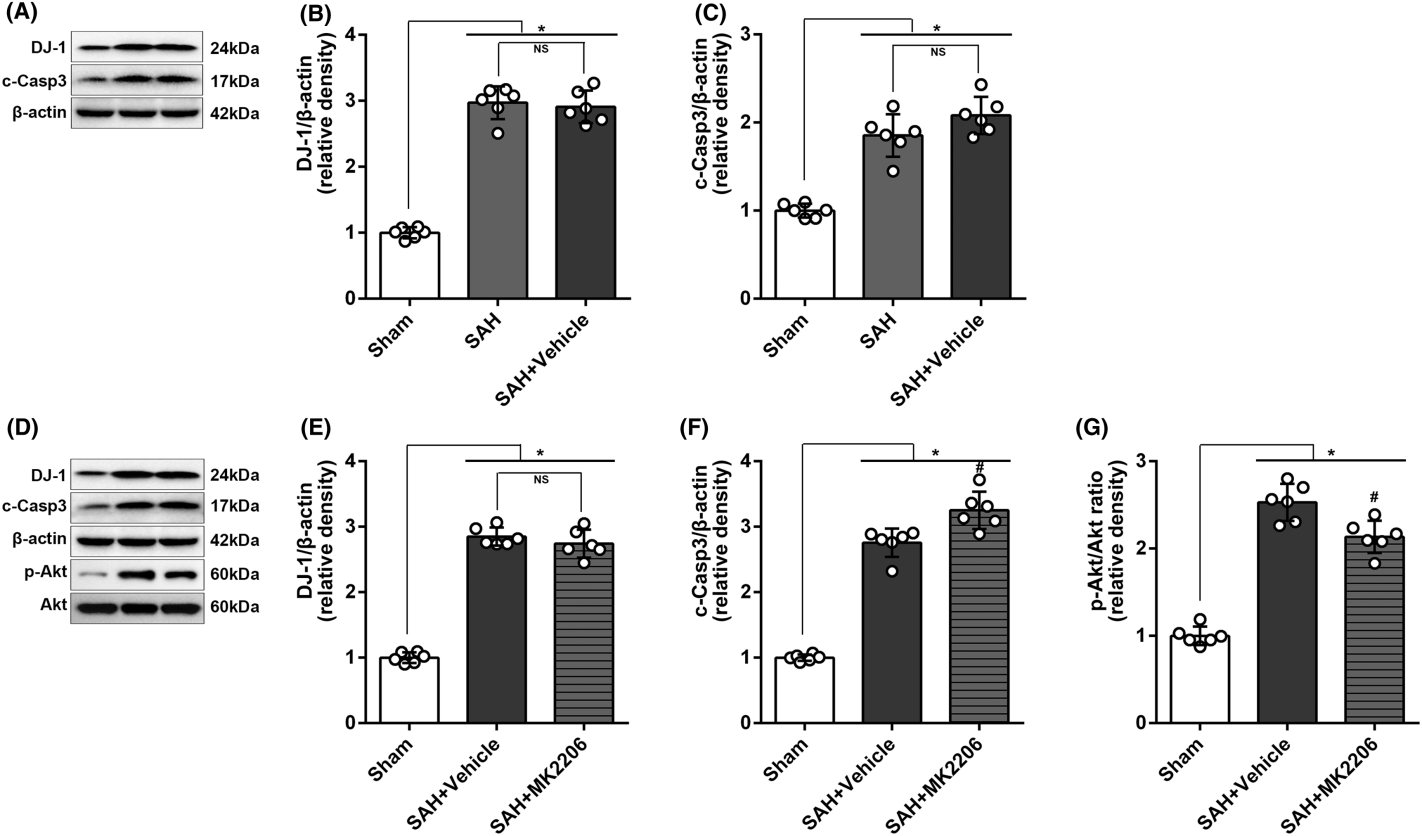
Representative bands and WB analyses. (A) Representative bands and the corresponding (B) DJ‐1 and (C) c‐Casp‐3 expression showed an increase after SAH. However, no difference was noted between SAH and vehicle group, one‐way ANOVA, *p < 0.05 versus sham, (*n* = 6/group). (D) Representative bands and expression of (E) DJ‐1, (F) c‐Casp‐3, and (G) p‐Akt/Akt ratio suggested that DJ‐1, c‐Casp‐3, and p‐Akt/Akt ratio increased significantly after SAH; the selective p‐Akt inhibitor, MK2206 effectively inhibited the p‐Akt and lead to the increase of c‐Casp‐3 but had no significant influence on DJ‐1, one‐way ANOVA, *p < 0.05 versus sham, #*p* < 0.05 versus vehicle. NS means statistical insignificance (*n* = 6/group).

WB band results (Figure 6A) showed that there was an increase in the expression of DJ‐1 (*p* < 0.05, Figure 6B) and c‐Casp‐3 (*p* < 0.05, Figure 6C) after SAH, and no difference noted between SAH and SAH + vehicle group. By using the selective Akt inhibitor, p‐Akt/Akt ratio was suppressed, and c‐Casp‐3 increased significantly. However, the expression of DJ‐1 seemed not to be influenced, indicating Akt played an antiapoptotic role in SAH and it was not upstream to DJ‐1 (Figure 6D–G). More likely, DJ‐1 is upstream to Akt.

#### 3.1.6 Mechanism of effect of betulin

**FIGURE 7:**
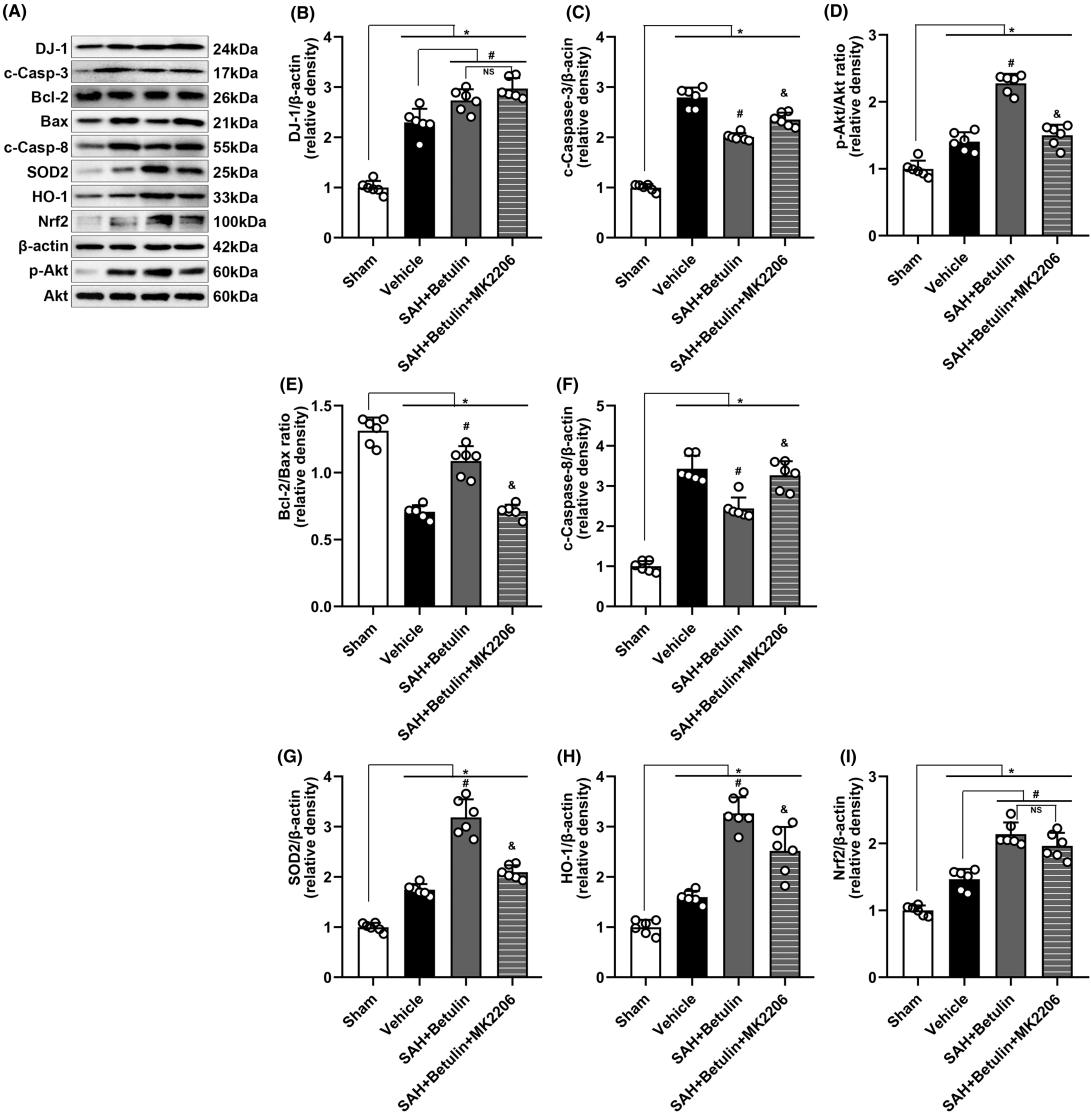
WB analysis to explore the mechanism. (A) Representative bands and the analysis of (B) DJ‐1, (C) c‐Casp‐3, (D) p‐Akt/Akt ratio, (E) Bcl‐2/Bax ratio, and (F) c‐Casp‐8 showed that the level of DJ‐1, p‐Akt/Akt ratio, c‐Casp‐3, and c‐Casp‐8 increased whereas that of Bcl‐2/Bax ratio decreased significantly, suggesting the intensified apoptosis after SAH onset. This could be reversed with betulin administration. However, MK2206 had no notable effect on DJ‐1 expression. In addition, (G) SOD2, (H) HO‐1, and (I) Nrf2 as well increased after SAH, and the expression could be strengthened by betulin. Likewise, the use of MK2206 reversed such effect but appeared no impact on Nrf2 expression. One‐way ANOVA, *p < 0.05 versus sham, #*p* < 0.05 versus vehicle, and *p* < 0.05 versus betulin. NS means statistical insignificance (*n* = 6/group). Betulin dose of 40 mg/kg was used in this study.

Representative bands of WB (Figure 7A) showed that DJ‐1, c‐Casp‐3, and p‐Akt/Akt increased (p<0.05, Figure 7B–D), Bcl‐2/Bax ratio decreased (*p* < 0.05, Figure 7E) while c‐Casp‐8 (*p* < 0.05, Figure 7F) as well increased significantly after SAH, which indicated that cell apoptosis is remarkable after SAH; the group administered with betulin had an increase in DJ‐1, p‐Akt/Akt, and Bcl‐2/Bax ratio while a significant decrease in c‐Casp‐3 and c‐Casp‐8. Thus, it suggested that betulin has an antiapoptotic effect. The use of Akt inhibitor, MK2206 could reverse these results except for the expression of DJ‐1, which implying that the antiapoptotic effect of betulin is through Akt‐dependent pathway and betulin could simultaneously boost the expression of DJ‐1, and Akt is downstream to DJ‐1. In the meantime, betulin treatment could significantly escalate the expression of antioxidative enzymes including SOD2 and HO‐1, and also the expression of Nrf2 (*p* < 0.05, Figure 7G–I). Likewise, this effect could be reversed with MK2206 but the expression of Nrf2 was not affected, suggesting Akt could upregulate the expression of SOD2 and HO‐1. However, it at least has no direct regulation on the expression of Nrf2.

#### 3.1.7 Mechanism study in vitro

**FIGURE 8:**
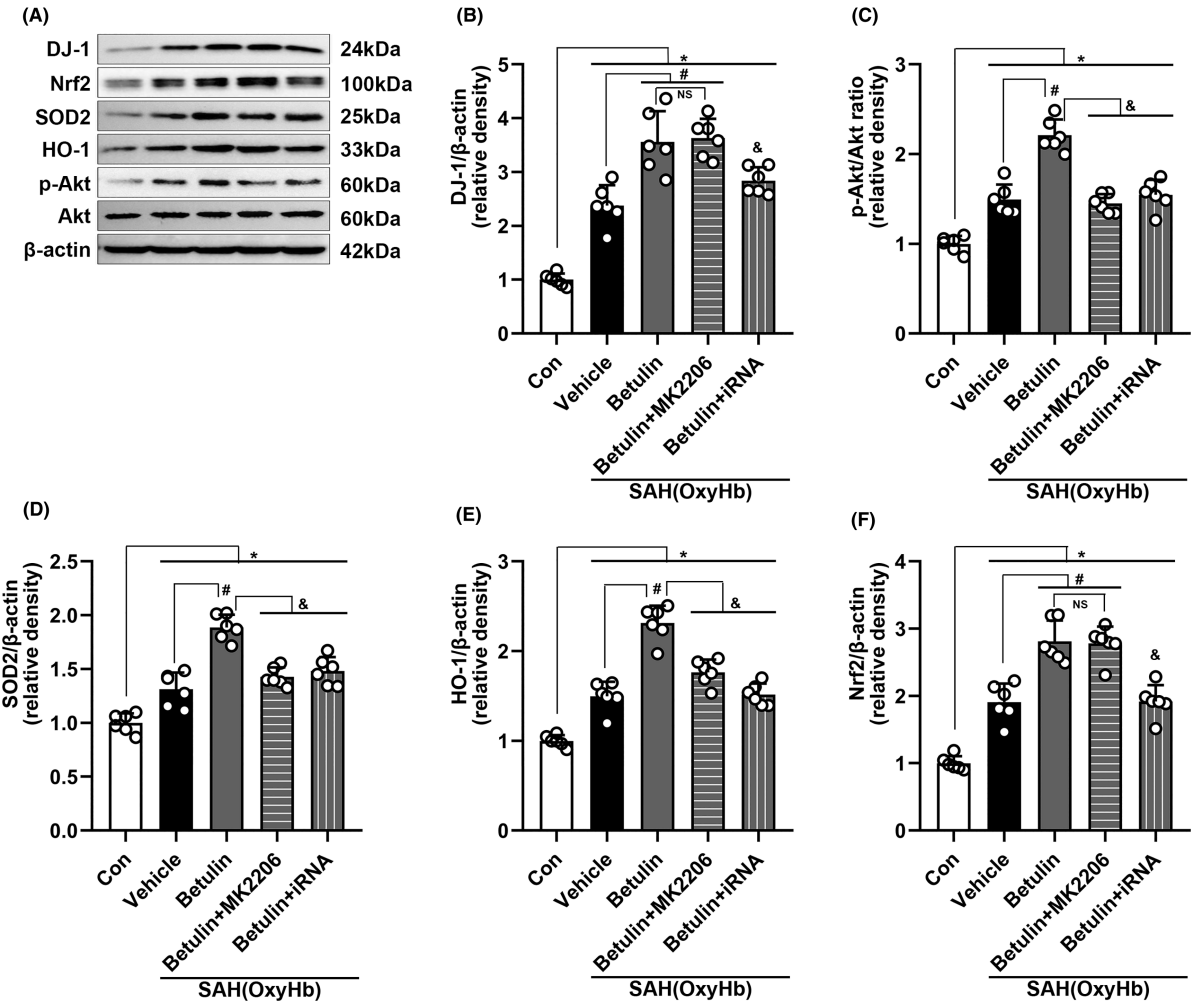
WB performed in vitro (SY5Y cells) and the analysis. (A) Representative bands and (B) DJ‐1, (C) p‐Akt/Akt ratio, (D) SOD2, (E) HO‐1, and (F) Nrf2 expression all increased significantly and could be further boosted by administering betulin. The boosting effect could be blocked with iRNA*Dj‐1*. The Akt inhibitor, MK2206 had similar effect except for on DJ‐1 and Nrf2, which was consistent with the in vivo study. One‐way ANOVA, *p < 0.05 versus sham, #*p* < 0.05 versus vehicle, and *p* < 0.05 versus betulin. NS means statistical insignificance (*n* = 6/group). Betulin dose of 20 μM was used in this study.

The results of protein bands (Figure 8A) and data analyses were consistent with the ones in vivo. The group treated with iRNA*Dj‐1* had significant reduction of DJ‐1, p‐Akt/Akt, SOD2, HO‐1, and Nrf2 expression (*p* < 0.05, Figure 8B–F), indicating that betulin exerts its effect through regulating DJ‐1 and the downstream Akt and Nrf2.

**FIGURE 9:**
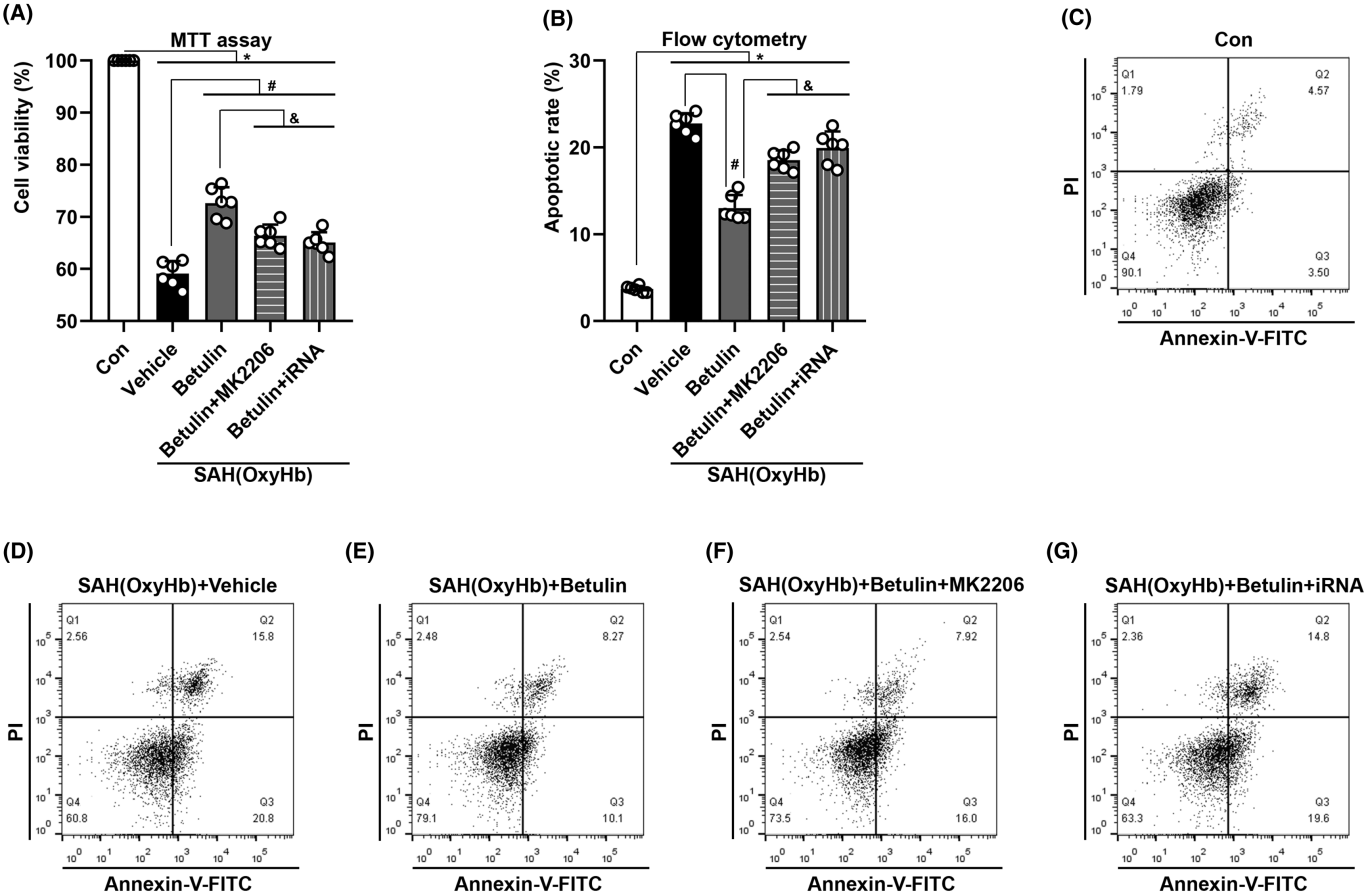
MTT assay and FCM technique. (A) MTT assay demonstrated massive cell deaths after SAH and the cell survival improved by betulin. This effect could be reversed using MK2206 or iRNA*Dj‐1*. (B) FCM calculation confirmed that after SAH induction, the neuronal apoptosis heightened; administration of betulin inhibited the apoptotic rate whereas it was erased by MK2206 or iRNA*Dj‐1*. Display of FCM images of (C) control group, (D) vehicle, (E) betulin, (F) betulin+MK2206, and (G) betulin + iRNA*Dj‐1* group. One‐way ANOVA, *p < 0.05 versus sham, #*p* < 0.05 versus vehicle, &*p* < 0.05 versus betulin. NS means statistical insignificance (*n* = 6/group).

Results from MTT assay proved that massive cell deaths occurred after SAH; administration of betulin could significantly promote cell survival. Nonetheless, this effect was reversed by Akt inhibitor or iRNA*Dj‐1* (*p* < 0.05, Figure 9A). Furthermore, FCM analysis confirmed that SY5Y cells undertook severe apoptosis after simulating SAH induction, and betulin could efficaciously rescue the process (*p* < 0.05, Figure 9B–G). Consistently, Akt inhibitor or iRNA*Dj‐1* prevented such protective effect and induced significant apoptosis, which convinced the function of DJ‐1/Akt signaling pathway.

## 4 DISCUSSION

There are increasing studies to report that DJ‐1 functions as a neuroprotective protein in various approaches, among which the relief of oxidative stress has been considered as the fundamental one and thus studied repeatedly.^33^, ^34^, ^35^ However, the favor of pro‐survival pathway by inducing antiapoptotic gene expression or recruiting survival proteins was less frequently reported. Moreover, the majority of present studies are confined to Parkinson's disease or ischemic stroke,^36^, ^37^, ^38^, ^39^ and typically carried out by means of in vitro experiments. Betulin is a chemical compound extracted from botanical components and has drawn much attention because of its multifarious functions such as antiapoptosis, anti‐inflammation, anticancer and antivirus.^18^ It was reported by a body of studies that betulin and its derivatives not only conferred benefit to various neurodegenerative diseases such as Parkinson's disease,^40^ Alzheimer Disease,^41^ but also offered protection in cerebral stroke, traumatic brain injury, hepatitis, and HIV infection.^18^ However, both betulin and DJ‐1 were lacking profound studies regarding hemorrhagic stroke involving SAH. We therefore set up the research to ascertain whether betulin and DJ‐1 had similar neuroprotection and correlation between each other in SAH. Our study results turned out to be affirmative, suggesting that betulin ameliorated the EBI and improved the prognosis after SAH in rats by upregulating the expression of DJ‐1. Precisely, betulin treatment at 1 h after SAH induction significantly reduced the gravity of neuronal apoptosis, shrunk brain edema, reduce oxidative stress, and systemically improved neurological outcomes.

Neuronal apoptosis was central to the pathological processes in EBI and was considered to be directly related to neurological deficits.^42^ It was noted by many studies that the neurons and astrocytes sustained apoptosis since the onset of SAH.^43^ Junn et al. reported that DJ‐1 could promote pro‐Akt survival pathway meanwhile impede the apoptosis.^44^ It was as well reported that DJ‐1 modulated Akt‐dependent pathway to promote cell survival.^45^ Previous studies suggested that both extrinsic and intrinsic mechanisms together with several caspases such as the initiators (caspase‐2,8,9,10) and the executioners (caspase‐3,6,7) were involved in apoptosis after SAH.^46^, ^47^, ^48^ Data from our study indicated that the increased intracellular DJ‐1 in turn upregulate p‐Akt, Bcl‐2, SOD2, and HO‐1 while decreased the levels of Bax, c‐Casp‐3, and c‐Casp‐8. We further cautiously analyzed the content of active caspase‐3, 8 and found that the two indices of apoptosis were suppressed in the SAH group treated with betulin but not the group with betulin plus Akt inhibitor (MK2206). The similar results were noted in betulin plus iRNA*Dj‐1* group only but the expression of Nrf2 was significantly inhibited. Thus, we have good reasons to interpret the consistent results as that the betulin treatment increases intracellular DJ‐1 which in turn levels up Akt to fulfill the antiapoptotic effect.

Oxidative injury is another key factor of EBI.^49^ An assortment of redox related enzyme such as SOD2, HO‐1, GSH, and TXNIP is associated with EBI after SAH.^50^ According to our previous works, drugs or proteins that could upregulate the antioxidative enzymes were simultaneously able to lessen the degree of BBB damage and brain edema.^51^ We examined and compared the level of SOD2 and HO‐1 between groups, and learned that they were increased in the betulin group but suppressed in the groups administered Akt inhibitor or iRNA*Dj‐1*, suggesting the antioxidative effect of betulin may be through DJ‐1/Akt‐dependent pathway. Actually, it had been verified by many studies that DJ‐1 did have antioxidative effect via the mechanism of ROS scavenging and antioxidative proteins recruiting. Given this, we deduce that betulin can boost intracellular DJ‐1 to play the antioxidative effect which contributes to brain edema mitigation and neurofunction improvement. The deduction was supported by the results BWC calculation and neurofunction evaluation.

Our study revealed that betulin has the effect of antiapoptosis, antioxidation, and alleviation of brain edema. Taken together, unsurprisingly it was consistent with the observations that the betulin‐treated group had statistically better neurological outcome. Our current data combined with previous evidence support the conclusion that betulin can exert neuroprotection on EBI after SAH through mechanisms of the DJ‐1/Akt‐dependent antiapoptotic and antioxidative pathways. However, there are still some obvious limitations in our work. First of all, we did not clarify the precise relationship between Akt and Nrf2. We indeed noted that iRNA*Dj‐1* could significantly downregulate the level of Nrf2, suggesting DJ‐1 could regulate the Nrf2 expression. The same effect was not found using Akt inhibitor, which suggested Akt might not affect the Nrf2 expression. In fact, some researchers reported that Akt inhibits the translocation of Nrf2 from mitochondria to the cytoplasm to play the antiapoptotic and antioxidative role.^52^, ^53^ Second, we explored the mechanism of betulin on apoptosis and oxidation though, the precise signaling pathways were not distinct. Third, the oxidative injury is no independent of apoptotic pathway. On the contrary, they have strong connection with each other and numerous signaling cross talk. Thus, more studies are needed to elucidate the signaling web and mechanisms. Lastly, we did not use the combination of selective agonist and antagonist of DJ‐1 to firmly prove its effectiveness on SAH. Also, the relationship between the natural compound betulin and its target innate proteins including DJ‐1 is of interest and needs to be further researched.

In summary, betulin treatment in the acute phase of SAH is capable of increasing intracellular DJ‐1 protein which in turn upregulates Akt to inhibit apoptosis and oxidative injury, which could ameliorate brain edema and improve neurological outcome. The treatment of betulin significantly attenuates the neurological deficits, and has the potential to benefit the patients suffering from SAH.

## AUTHORS CONTRIBUTIONS

XL, SY, and QL brewed the idea and initiated the manuscript; YZ and ZC collected and analyzed the data; HW and TL revised the manuscript and supervised the research and shared correspondence.

## FUNDING INFORMATION

This work was funded by the National Natural Science Foundation of China (Grant No. 82001278), The Fund for Young Doctors with the First People's Hospital of Yunnan Province (Grant No. KHBS‐2022‐018), Joint Projects of Yunnan Provincial Science and Technology Department and Kunming Medical University for Applied Basic Research (Grant No. 202101AY070001‐252), Yunnan Fundamental Research Projects (Grant No. 202401CF070007), Joint Projects of Yunnan Provincial Science and Technology Department and Kunming University of Science and Technology (Grant No. KUST‐KH2023005Z), and The Innovation Research Projects on Cerebrovascular Diseases for Young Doctors, China International Medical Foundation (Z‐2016‐20‐2101).

## CONFLICT OF INTEREST STATEMENT

The authors have no conflicts of interest to declare.

## Supporting information


Figure S1.

Table S1.

Data S1.

## Data Availability

All data of this study are available from the corresponding authors, and agreed by all the authors to share under reasonable request.
