# Supplementary material for: Betulin ameliorates neuronal apoptosis and oxidative injury via DJ‐1/Akt/Nrf2 signaling pathway after subarachnoid hemorrhage
Source: CNS Neurosci Ther. 2024 Sep 5;30(9):e70019. doi: 10.1111/cns.70019 (PMC11377304; doi:10.1111/cns.70019)
Supplement: Supplementary file 1 — Figure S1. [file CNS-30-e70019-s002.docx]

**Supplemental Figures**


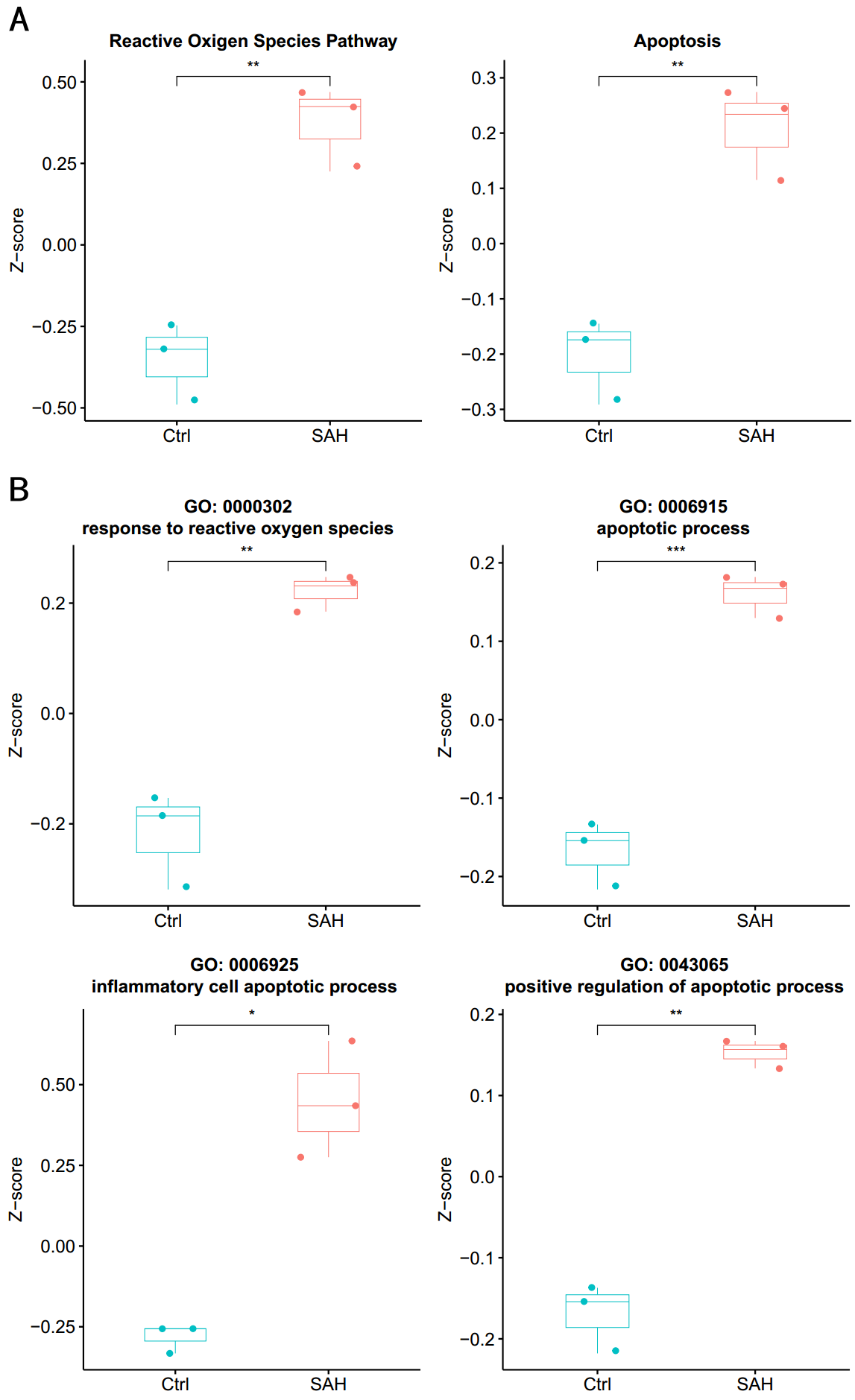


**Supplemental Figure 1.**

(A) Gene set variation analysis scores of apoptosis and reactive oxygen species (ROS) pathway. (B) Gene ontology enrichment of response to ROS and apoptotic process.


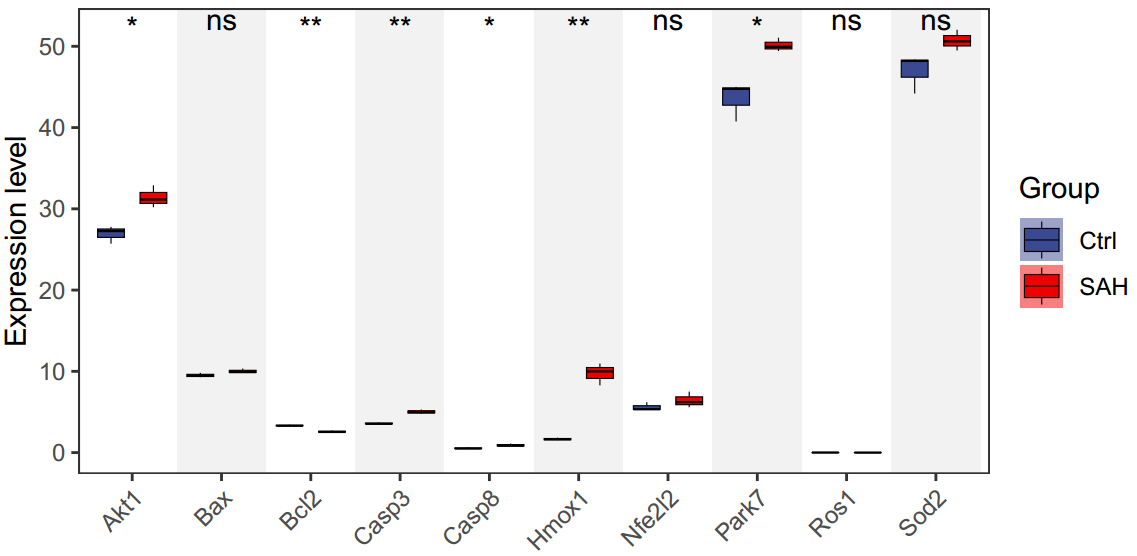


**Supplemental Figure 2.**

Box plot showing 10 genes expression levels of DJ-1 and its downstream signaling pathways.
