# Supplementary material for: Betulin ameliorates neuronal apoptosis and oxidative injury via DJ‐1/Akt/Nrf2 signaling pathway after subarachnoid hemorrhage
Source: CNS Neurosci Ther. 2024 Sep 5;30(9):e70019. doi: 10.1111/cns.70019 (PMC11377304; doi:10.1111/cns.70019)
Supplement: Supplementary file 2 — Table S1. [file CNS-30-e70019-s001.docx]

| **Groups** | **Mortality** | **Excluded** |
| --- | --- | --- |
| **Experiment 1: the expression changes of DJ-1** |  |  |
| Sham (n=8) | 0 (0/8) | 0 |
| SAH (3h, 6h, 12h, 24h, 72h) (n=33) | 3.0% (1/33) | 2 |
| **Experiment 2.1: short-term outcome study (24h and 72h)** |  |  |
| Sham (n=24) | 0 (0/24) | 0 |
| SAH+Vehicle (n=27) | 11.1% (3/27) | 1 |
| Betulin 20mg/kg (n=26) | 7.7% (2/26) | 0 |
| *Betulin 40mg/kg (n=24) | 0 (0/24) | 0 |
| Betulin 80mg/kg (n=24) | 0 (0/24) | 2 |
| **Experiment 2.2: long-term outcome study** |  |  |
| Sham (n=8) | 0 (0/8) | 0 |
| SAH+Vehicle (n=9) | 11.1% (1/9) | 0 |
| SAH+GPNMB (n=9) | 11.1% (1/9) | 0 |
| **Experiment 3: signaling pathway exploration** |  |  |
| Sham (n=6) | 0 (0/6) | 0 |
| SAH+Vehicle (n=7) | 14.3% (1/7) | 0 |
| SAH+Betulin (n=6) | 0 (0/6) | 0 |
| SAH+Betulin+MK2206 (n=8) | 25% (2/8) | 0 |
| **TOTAL** |  |  |
| Sham | 0 (0/46) | 0 |
| SAH | 6.4% (11/173) | 5 |

**Table 1 Grouping and use of rats in the studies**

*Betulin 40mg/kg was the minimal-effective dose and adopted for the following studies.

A total of 224 rats were used: 46 in sham group, 173 in SAH group. Plus, 5 rats were excluded due to fail to establish the SAH model.
