# Supplementary material for: Betulin ameliorates neuronal apoptosis and oxidative injury via DJ‐1/Akt/Nrf2 signaling pathway after subarachnoid hemorrhage
Source: CNS Neurosci Ther. 2024 Sep 5;30(9):e70019. doi: 10.1111/cns.70019 (PMC11377304; doi:10.1111/cns.70019)
Supplement: Supplementary file 3 — Data S1. [file CNS-30-e70019-s003.pdf]

Uncropped Gel Images

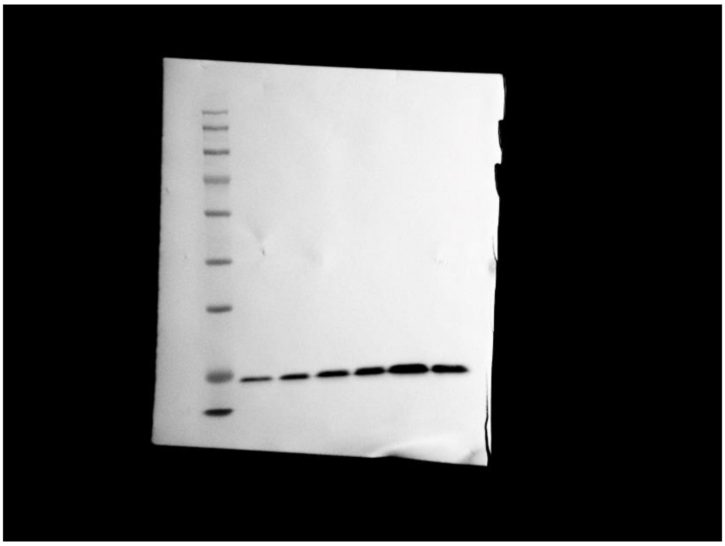

Figure 1 DJ-1

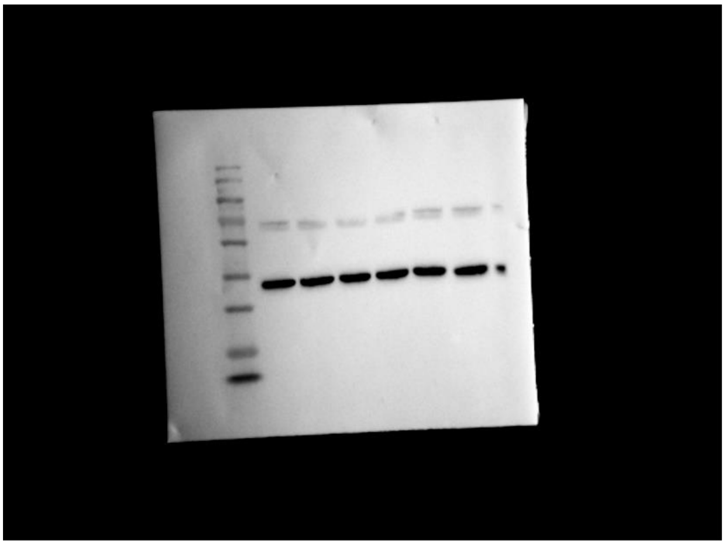

Figure 1 β-actin

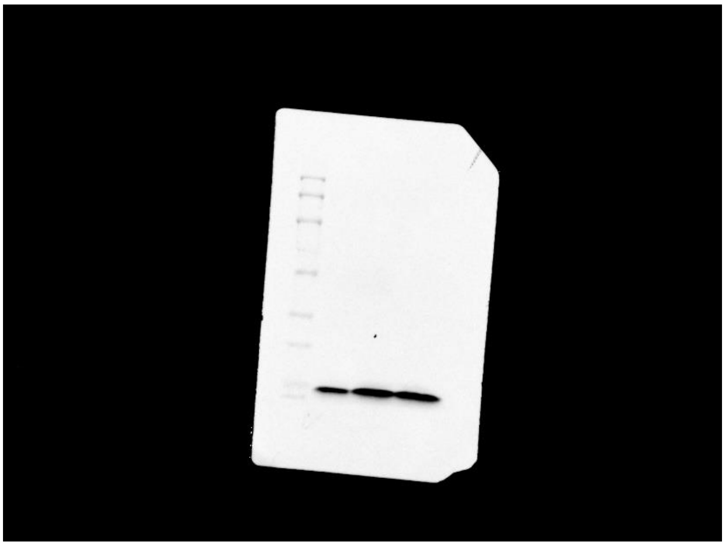

Figure 6A DJ-1

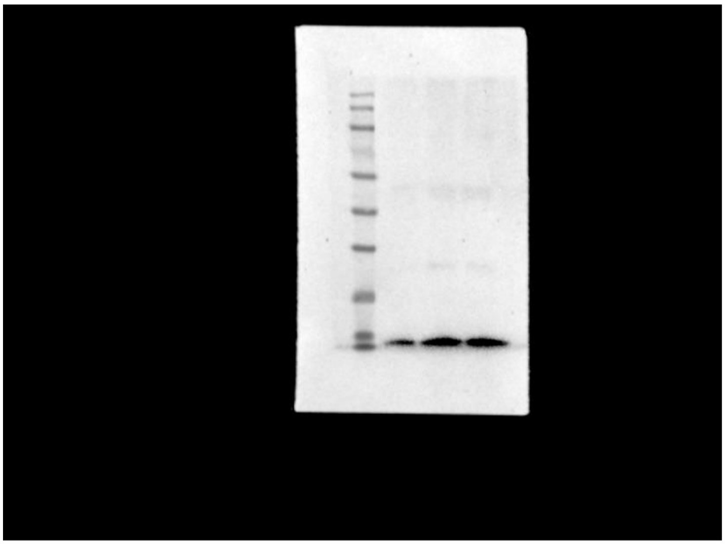

Figure 6A c-Casp-3

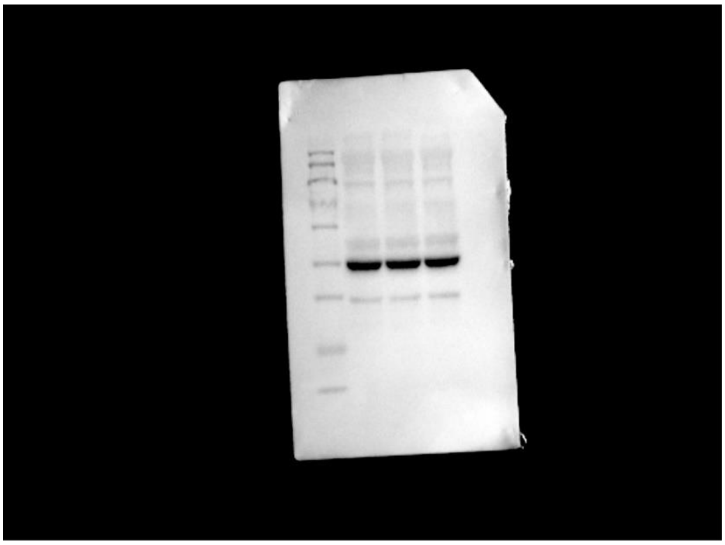

Figure 6A β-actin

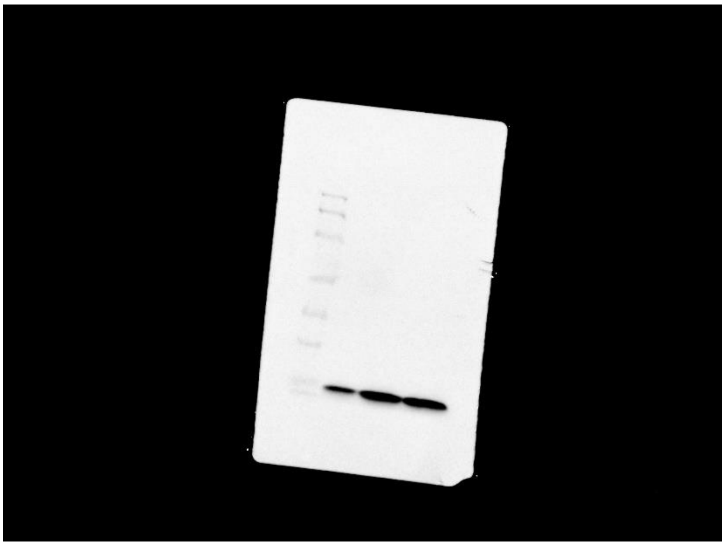

Figure 6D DJ-1

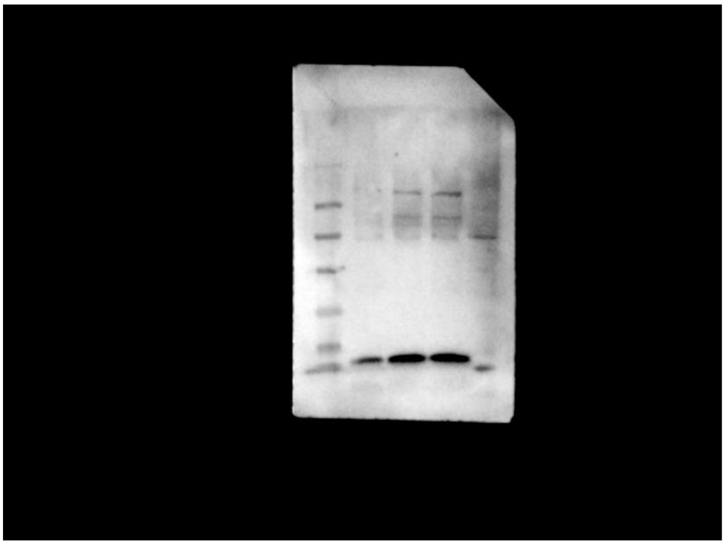

Figure 6D c-Casp-3

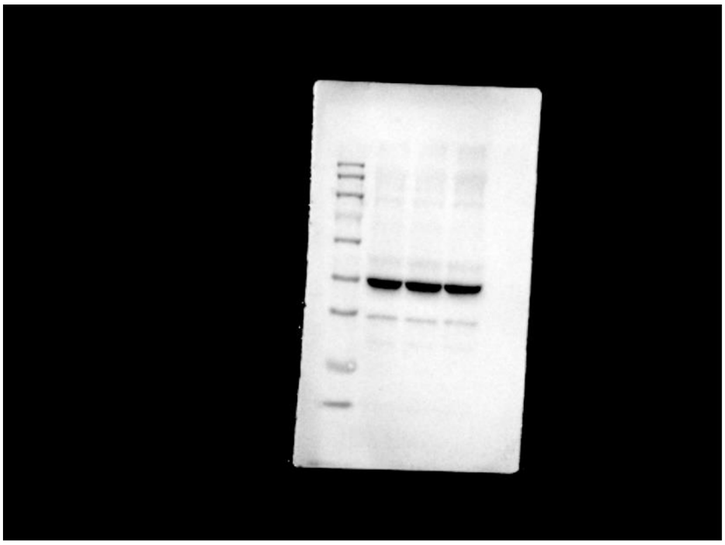

Figure 6D β-actin

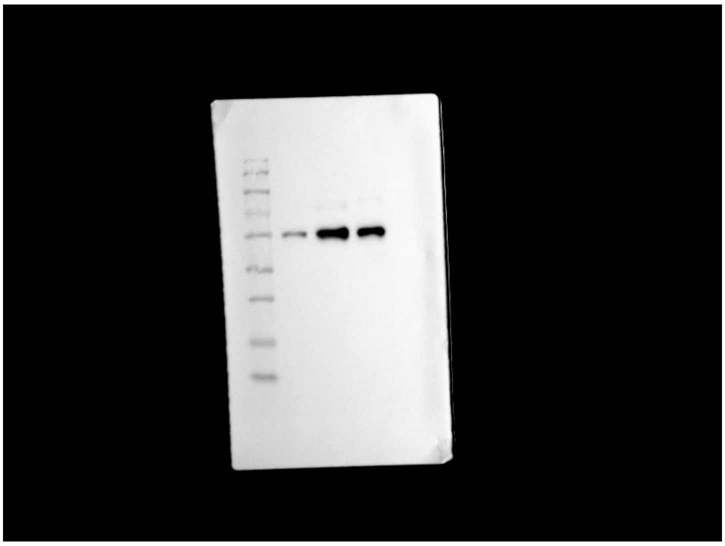

Figure 6D p-Akt

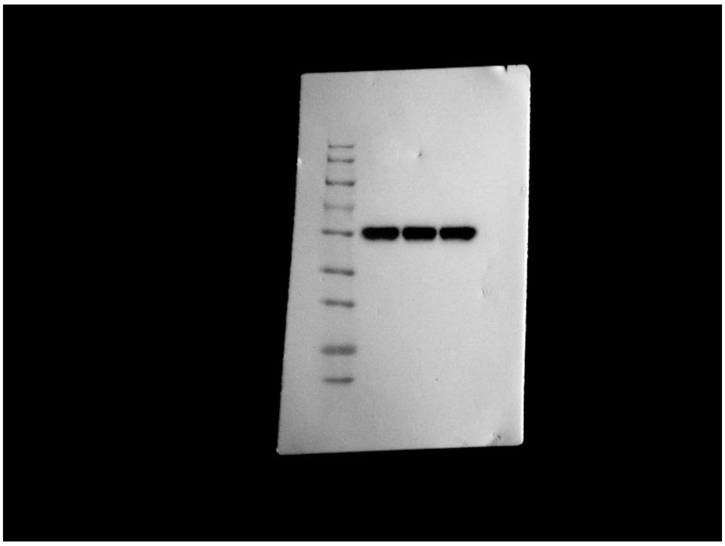

Figure 6D Akt

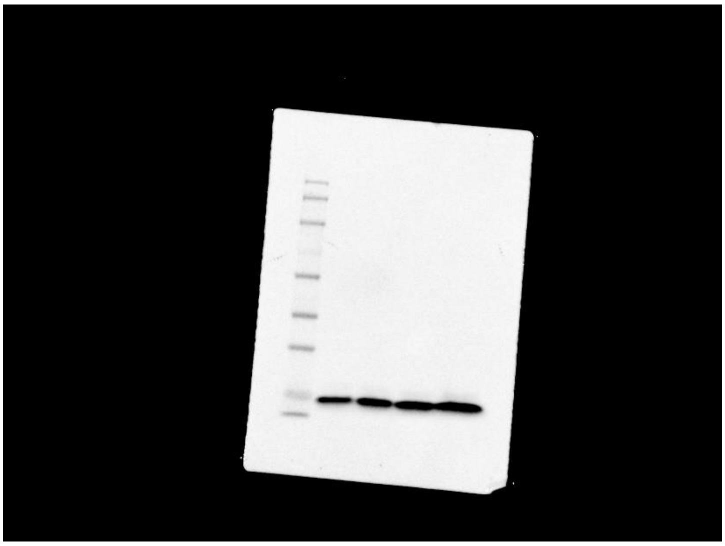

Figure 7A DJ-1

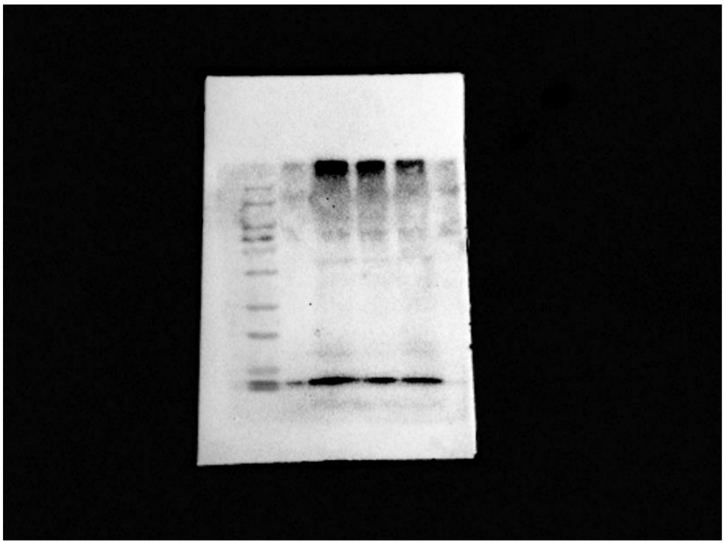

Figure 7A c-Casp-3

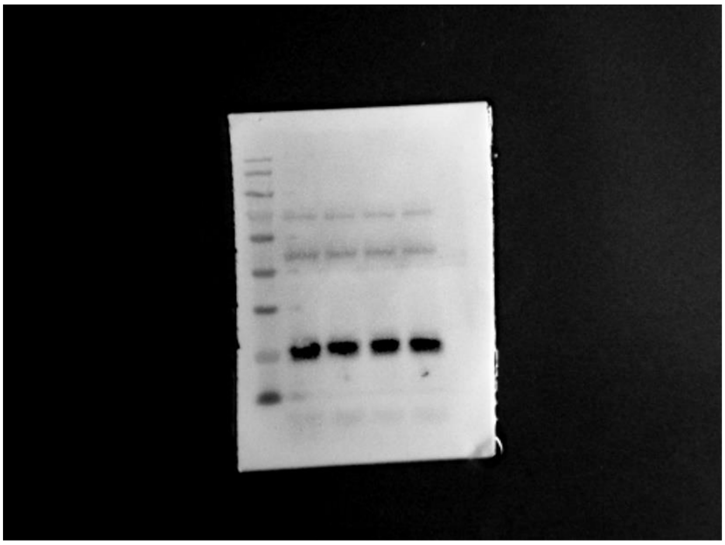

Figure 7A Bcl-2

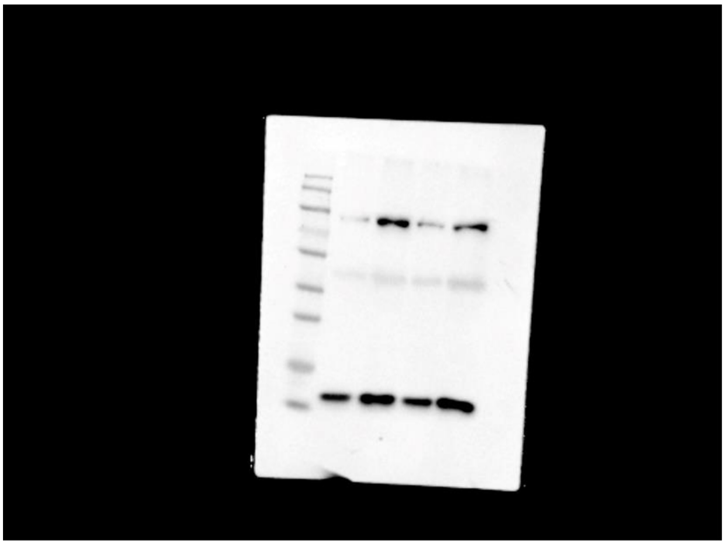

Figure 7A Bax

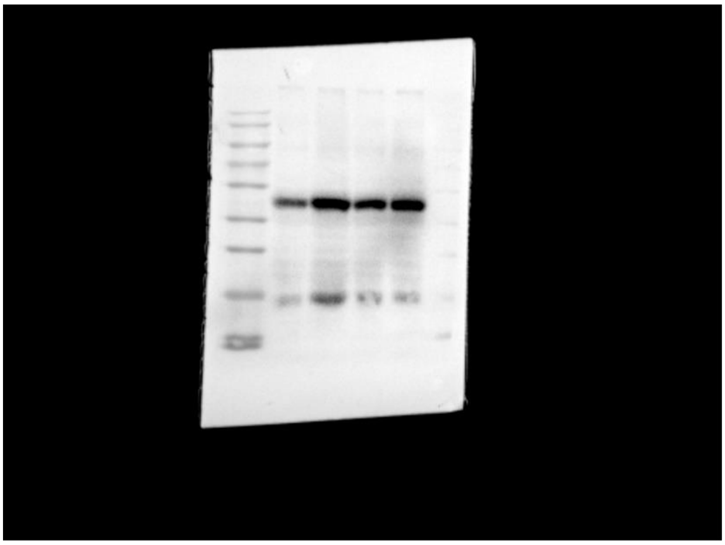

Figure 7A c-Casp-8

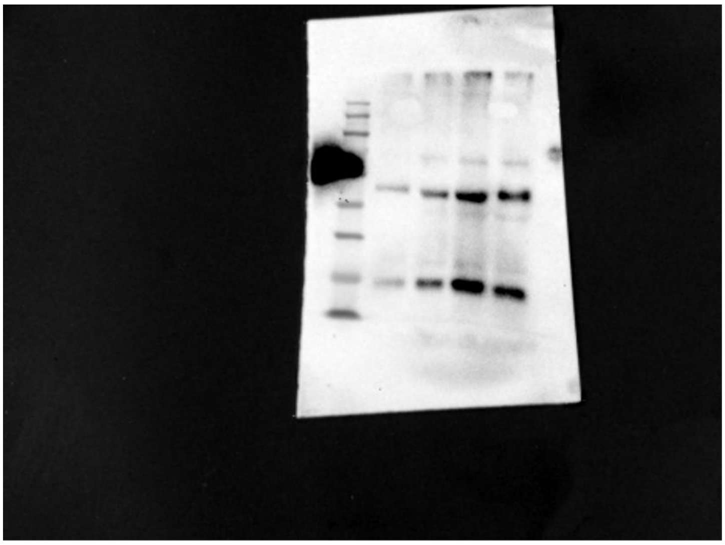

Figure 7A SOD2

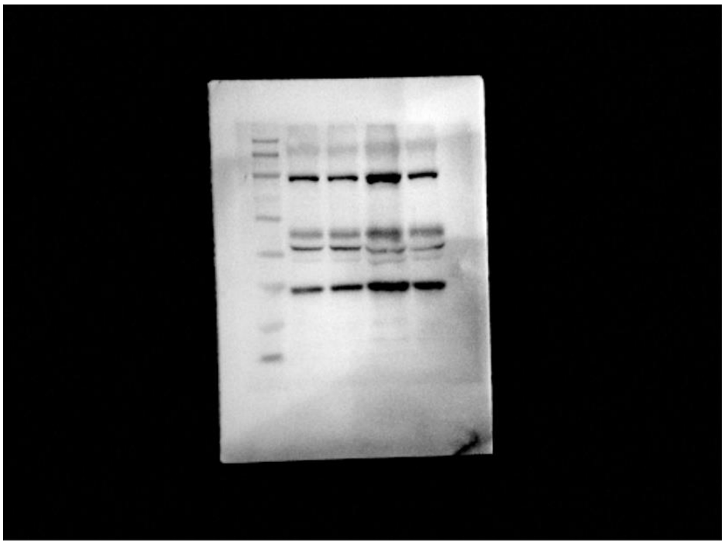

Figure 7A HO-1

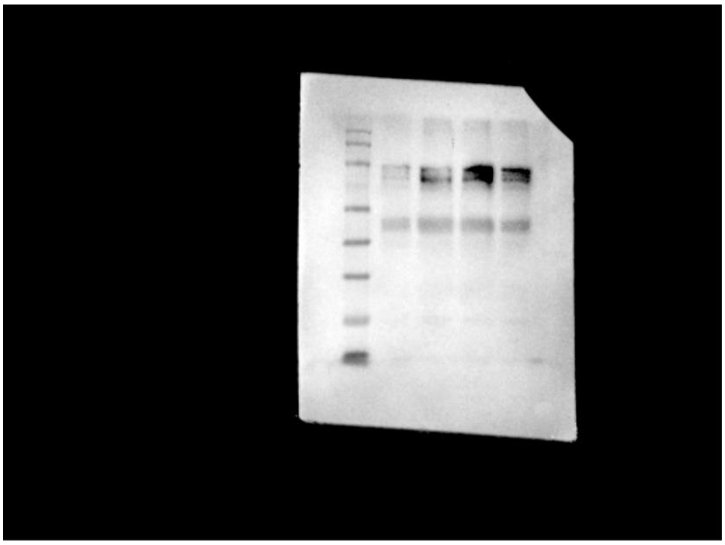

Figure 7A Nrf2

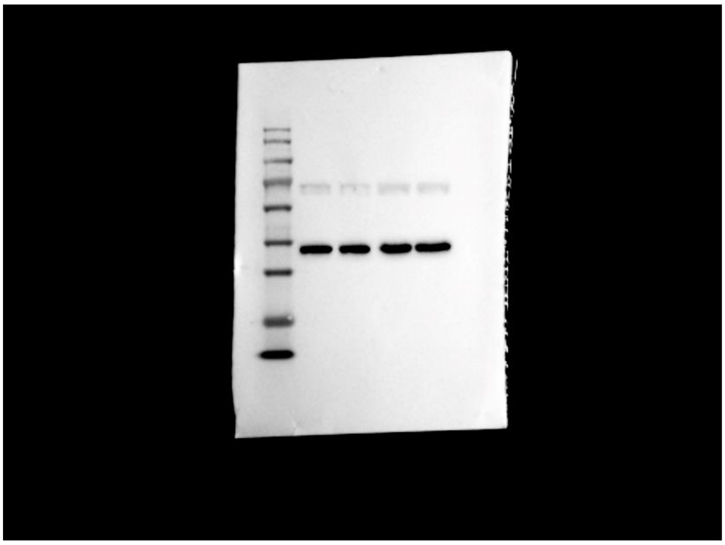

Figure 7A β-acin

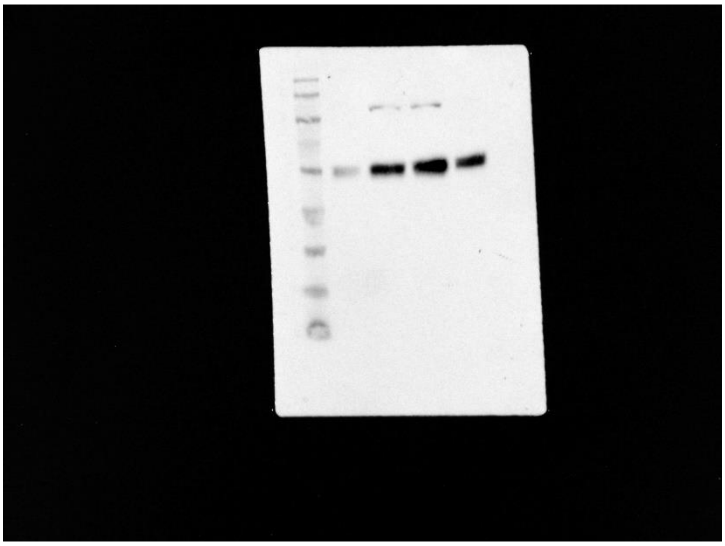

Figure 7A p-Akt

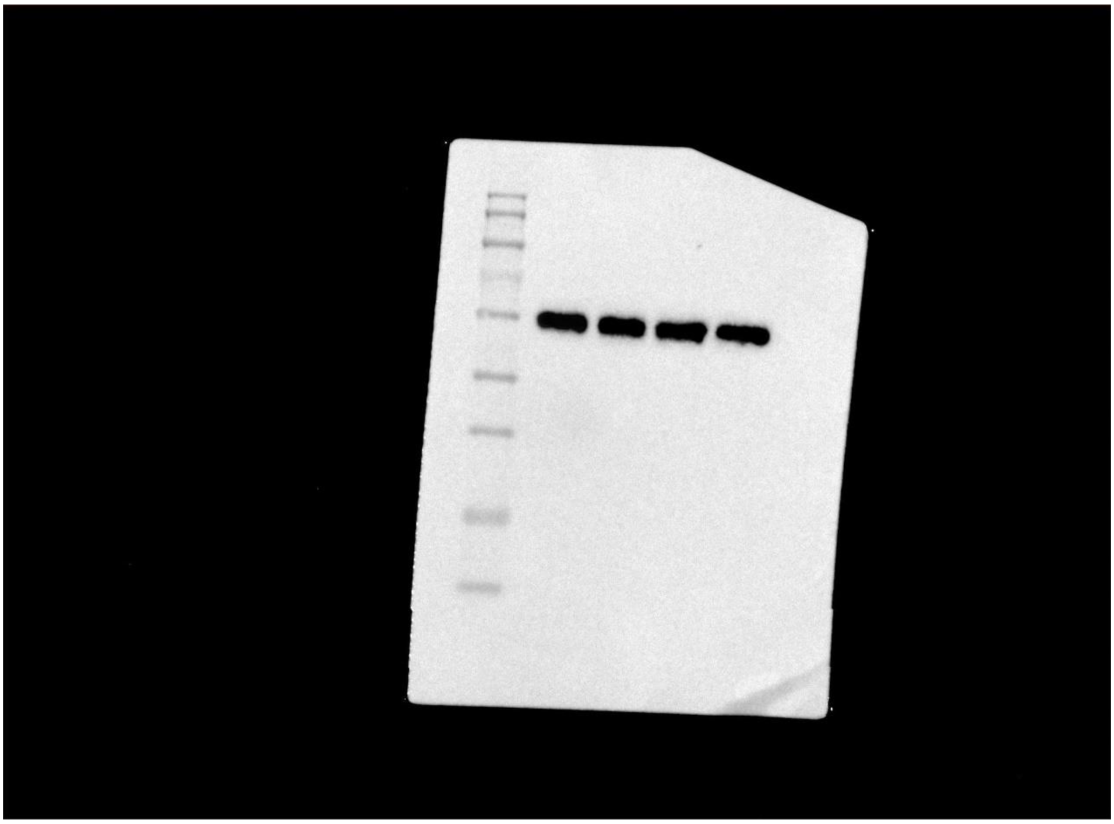

Figure 7A Akt

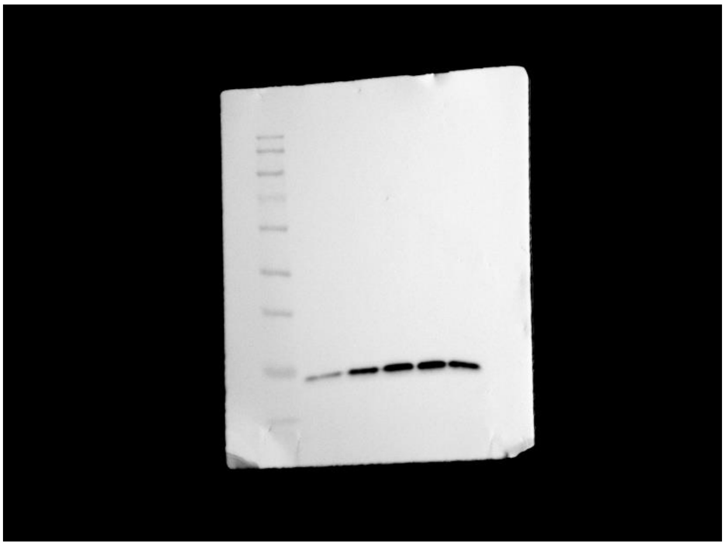

Figure 8A DJ-1

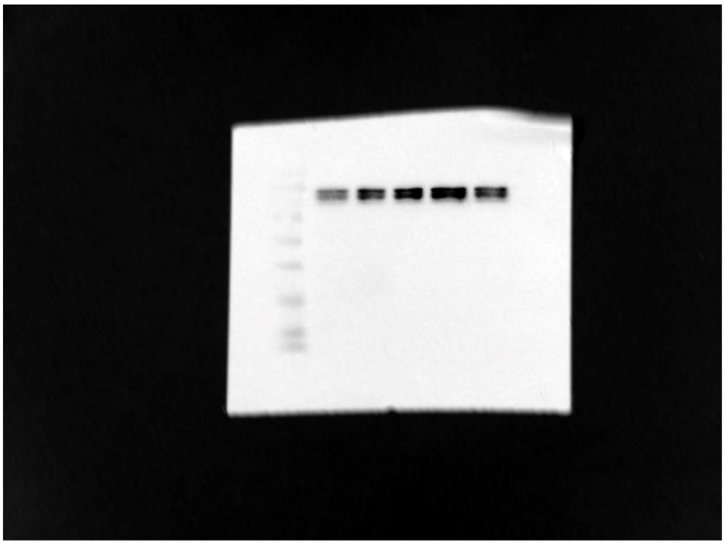

Figure 8A Nrf2

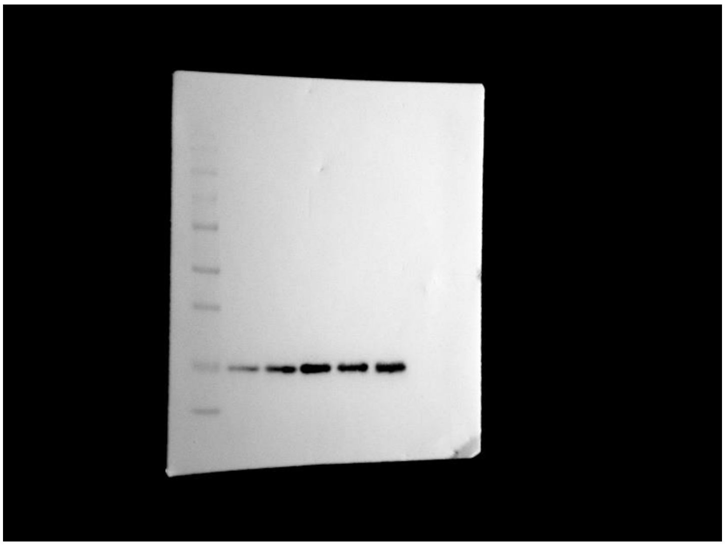

Figure 8A SOD2

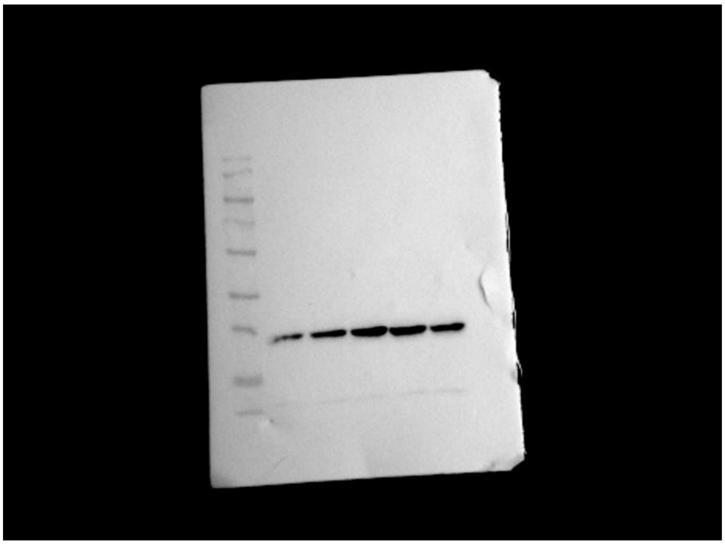

Figure 8A HO-1

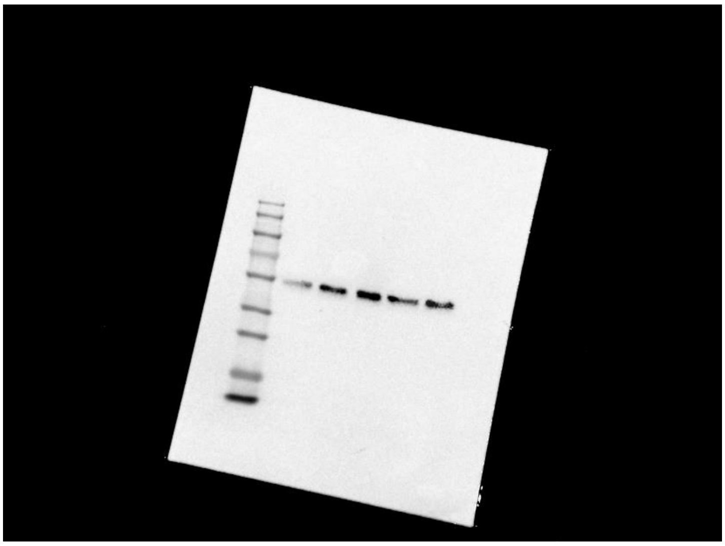

Figure 8A p-Akt

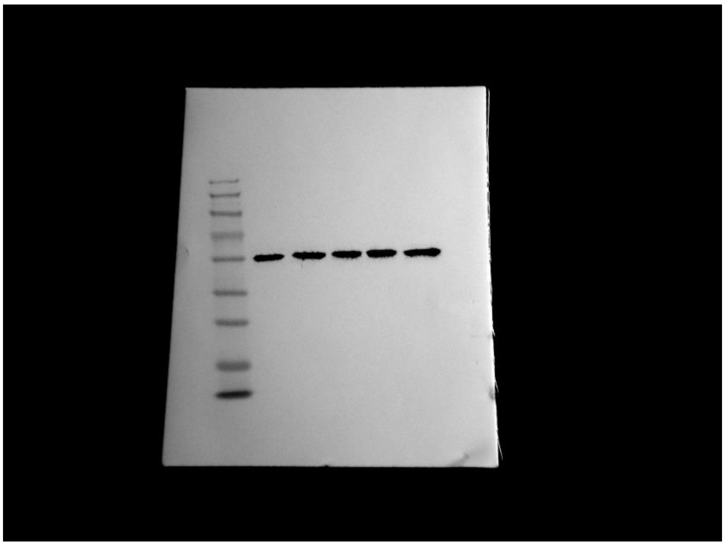

Figure 8A Akt

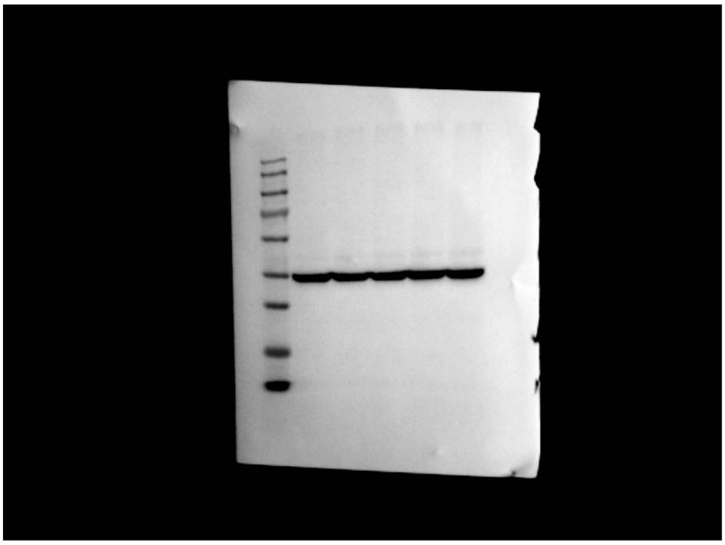

Figure 8A β-actin
